# Discrimination of 2D wall textures by passive echolocation for different reflected-to-direct level difference configurations

**DOI:** 10.1371/journal.pone.0251397

**Published:** 2021-05-27

**Authors:** Léopold Kritly, Yannick Sluyts, David Pelegrín-García, Christ Glorieux, Monika Rychtáriková

**Affiliations:** 1 Research Department of Architecture—Building and Room Acoustics, Faculty of Architecture, KU Leuven, Brussel, Belgium; 2 EPF–Graduate School of Engineering, Sceaux, France; 3 ZMB Lab. of Acoustics, Department of Physics and Astronomy, KU Leuven, Heverlee, Belgium; 4 Faculty of Civil Engineering, STU Bratislava, Bratislava, Slovakia; US Department of Agriculture, UNITED STATES

## Abstract

In this work, we study people’s ability to discriminate between different 2D textures of walls by passive listening to a pre-recorded tongue click in an auralized echolocation scenario. In addition, the impact of artificially enhancing the early reflection magnitude by 6dB and of removing the direct component while equalizing the loudness was investigated. Listening test results for different textures, ranging from a flat wall to a staircase, were assessed using a 2 Alternative-Forced-Choice (2AFC) method, in which 14 sighted, untrained participants were indicating 2 equally perceived stimuli out of 3 presented stimuli. The average performance of the listening subjects to discriminate between different textures was found to be significantly higher for walls at 5m distance, without overlap between the reflected and direct sound, compared to the same walls at 0.8m distance. Enhancing the reflections as well as removing the direct sound were found to be beneficial to differentiate textures. This finding highlights the importance of forward masking in the discrimination process. The overall texture discriminability was found to be larger for the walls reflecting with a higher spectral coloration.

## Introduction

Echolocation is originally known in the context of animals such as bats and dolphins orienting themselves and recognizing their environment by interpreting echoes from self-produced high frequency burst sequences and chirps, mostly outside of the audible frequency range of human beings [1, 2]. This orientation technique allows them to locate nearby obstacles, predators and preys with a fine spatial resolution and accuracy [3, 4]. This method, also called biosonar, is based on listening to self-emitted sounds along with the reflection patterns induced by the surroundings [5].

Although the echolocation technique is typically used by animals, it can also be beneficial to people, especially when it comes to visually challenging conditions, in which acoustic feedback of the environment allows for mapping its features without only relying on visual cues. In this context, echolocation is highly valuable for blind individuals in adaptation to vision loss especially in combination with the use of a long cane [6].

In spite of the extraordinary power of the human auditory system, most people are not aware of these beneficial auditive possibilities and focus on the exploration of visual cues, which are often a prerequisite for our mobility, identification and information acquisition. Visually impaired persons or blind people are forced to use non-visual methods to navigate in their environment, either based on haptic or acoustic cues.

The use of other senses than sight (i.e., tactile and auditory senses) for the orientation of blind people has been investigated in holistic approaches [7] in which spaces needed to be perceived through a multisensorial approach. The use of non-visual cues for navigation has also been explored by Dodsworth et al. [8] and others (see [9–12]). In this framework, the project “Designing in the Dark” was conducted by letting sighted people experience the effect of visual impairment on the performance of navigation tasks, as well as including impaired end-users of spaces within their design [13].

Acoustic echolocation is mostly found under 3 forms: passive echolocation, active echolocation and sensory substitution. Passive echolocation consists in using external sounds or noise to map the environment. Active echolocation makes use of self-triggered sound, using a portable clicking device or cane hits; or self-produced stimuli, by generating oral clicks and hisses. The latter sounds are the two most used among the echolocator community. Oral clicks, also called tongue or palatal clicks, look like a Bessel pulse [14] and are the most commonly used stimuli by echolocators providing a high performance to perform echolocation task [14–16], due to their impulsive nature (Full Width Half Maximum envelope duration of about 3ms) and high sound pressure level (sound exposure level 35-65dB) [17]. Hisses, due to their wide spectrum (>5kHz) and long duration (order of 1 second or more) are particularly efficient for short range detection, by exploiting the spectral coloration of the perceived sound, which is caused by frequency dependent interference between reflected and direct sound [18, 19] varying proportionally to the source bandwidth [20]. Alternatively, Sensory Substitution Devices (SSDs) can provide, through haptic or auditory feedback, information on nearby obstacles acquired using ultrasound or optic signals [2, 21]. Some other assistive devices make echolocation accessible to users, regardless of their ability to self-produce high performance stimuli, i.e. of high sound intensity and high peak frequencies, by generating them on an external acoustic source mounted on the head of the echolocator [22]. In the blind community, opinions on the added value of SSDs are not unanimous, with objections mostly related to the undesirable need for external electrical power to operate, as well as to their cost and salient design [23]. Each of these echolocation forms can be learned and developed by dedicated training on both visually impaired and sighted people having normal hearing [24]. The learning of echolocation by training is often stressed by its users [9, 25], to highlight that most people can, to some extent, successfully echolocate if they commit sufficient training time and effort.

Although human echolocation is typically associated with obstacle detection and location, the spectrotemporal content of acoustic echoes can potentially also provide information on geometrical features of reflecting objects. In 1996, Beranek [26] mentioned the possibility to extract, from sound, information on the texture of a wall, via “the subjective impression that listeners derive from the patterns in which the sequence of early sound reflections arrive at their ears”. In room acoustic assessments, optimizations and simulations, the texture of the walls are taken into account via the scattering or diffusion coefficient [27]. An increase of the scattering coefficient of walls inside a room has been reported to cause an increase of the early decay time (EDT) and reverberation time (T30), and accordingly a decrease of clarity (C80) [27, 28]. The perception of the effects of a changed scattering coefficient is strongly influenced by the choice of excitation stimulus as well as by the location of the listener inside the room [28, 29]. In the framework of concert hall simulations, it has been assessed that differences in scattering coefficient larger than 0.4 compared to a room with a scattering coefficient of 0.9 are audible [27]. Changes in surface scattering properties primarily affect the frequency spectrum of a sound [30] and the perception of its spaciousness [31]. In a recent round robin test, finding an adequate simulation algorithm for scattering in ray-based softwareturned out to be challenging, and simplification of scattering effects in acoustic simulation software led to audible artefacts [32]. Experiments demonstrating the practical use of echolocation to recognize architectural features have been presented in Refs. [33, 34]. An investigation on the precedence effect and its relation to surface textures in realistic conditions was done by Robinson et al. [35].

In the following, we report on the audibility of differences in auralized click sound generated and heard by an echolocator in the neighbourhood of different walls (one of them shaped as a staircase), reflecting and/or scattering a part of the sound back to the echolocator, in a virtual environment. Two cases are considered: one with the wall nearby the echolocator, so that the reflected sound overlaps with the direct sound, and another one with the wall further away, so that there is no overlap between the direct and reflected sounds. Two different concepts of coloration are considered: one related to the comb filtering that results from the interaction between the direct and reflected sound, and another due to the spectral shaping and fine time distribution of the reflections. The audibility is assessed in ABX-type listening tests in which a test person is asked which of two sounds, A or B, equals a third sound X. In the following, we first describe the click stimulus as well as the features of the different obstacles used in the auralization. Next, details on the ABX procedure are provided, and a statistical analysis of the listening test results that were performed on 14 test persons is presented. Finally, conclusions and related perspectives for future contributions are drawn.

## Method

### Stimuli

#### Room impulse response computation

**Fig 1:**
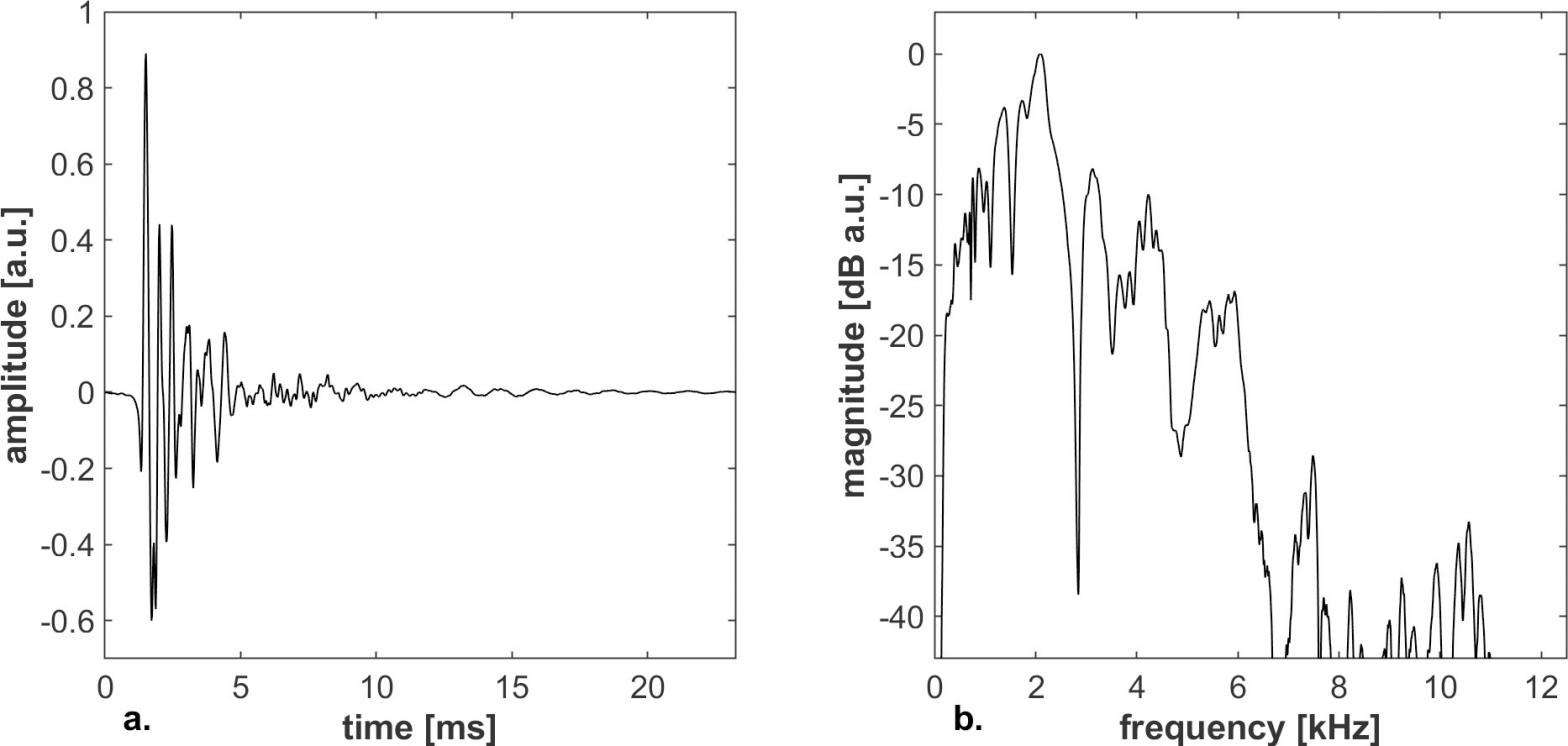
Characterization of a palatal click. The representations are respectively a temporal (a) and a spectral (b) plot of the tongue click used in this study. The spectrum was computed by Fourier transform without windowing.

**Fig 2:**
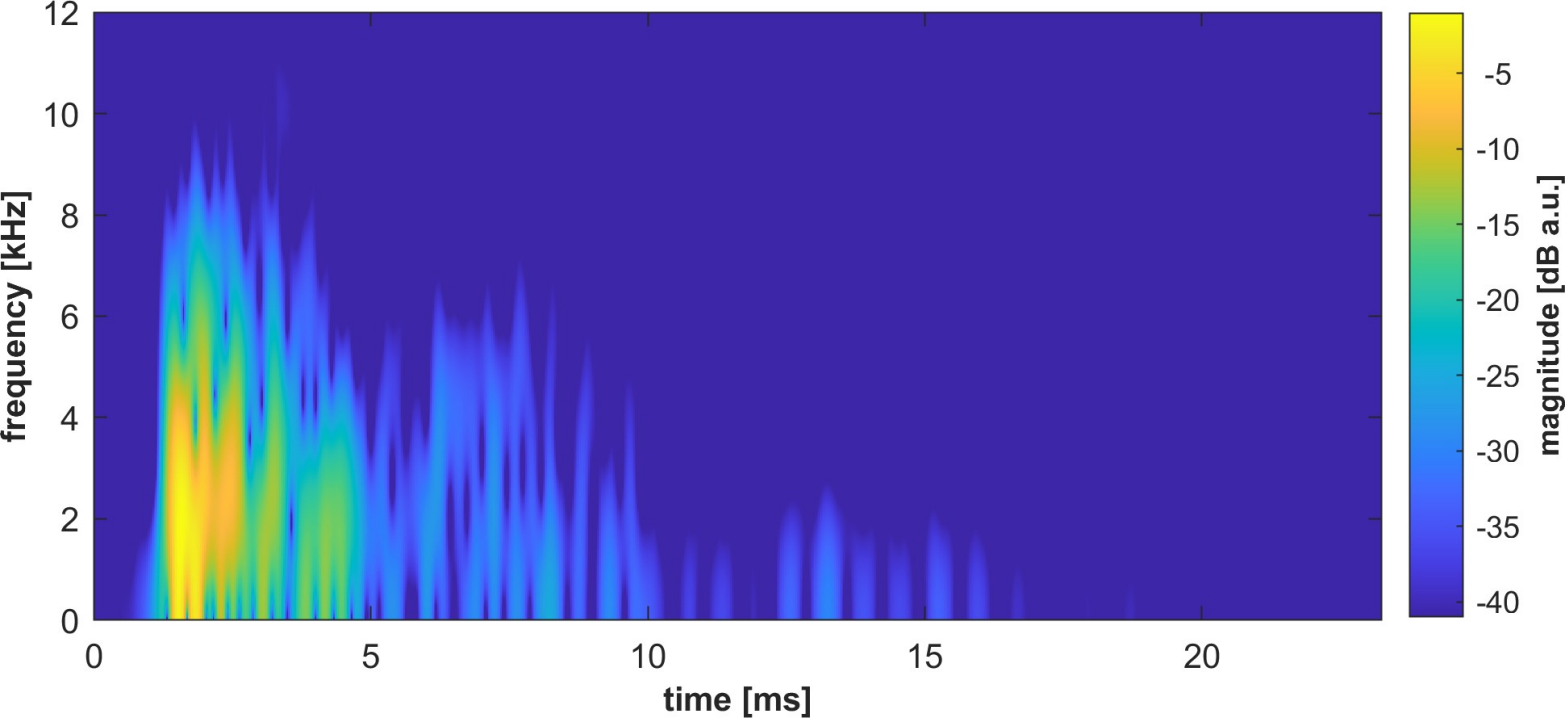
Continuous wavelet transform of a tongue click. The duration of the wavelets was set to 7 samples at 44.1kHz, i.e. 159μs. The spectro-temporal representation was computed up to 12kHz and was normalized in magnitude.

Room Impulse Responses (RIRs) of six different textures were simulated by a 2D Finite Difference calculation, using a spherically symmetric Gaussian source of 2.8cm radius, sized to match the mouth opening of an echolocator performing a tongue click, thereby generating frequencies up to 13kHz [see [19] and Figs 1 and 2].

**Fig 3:**
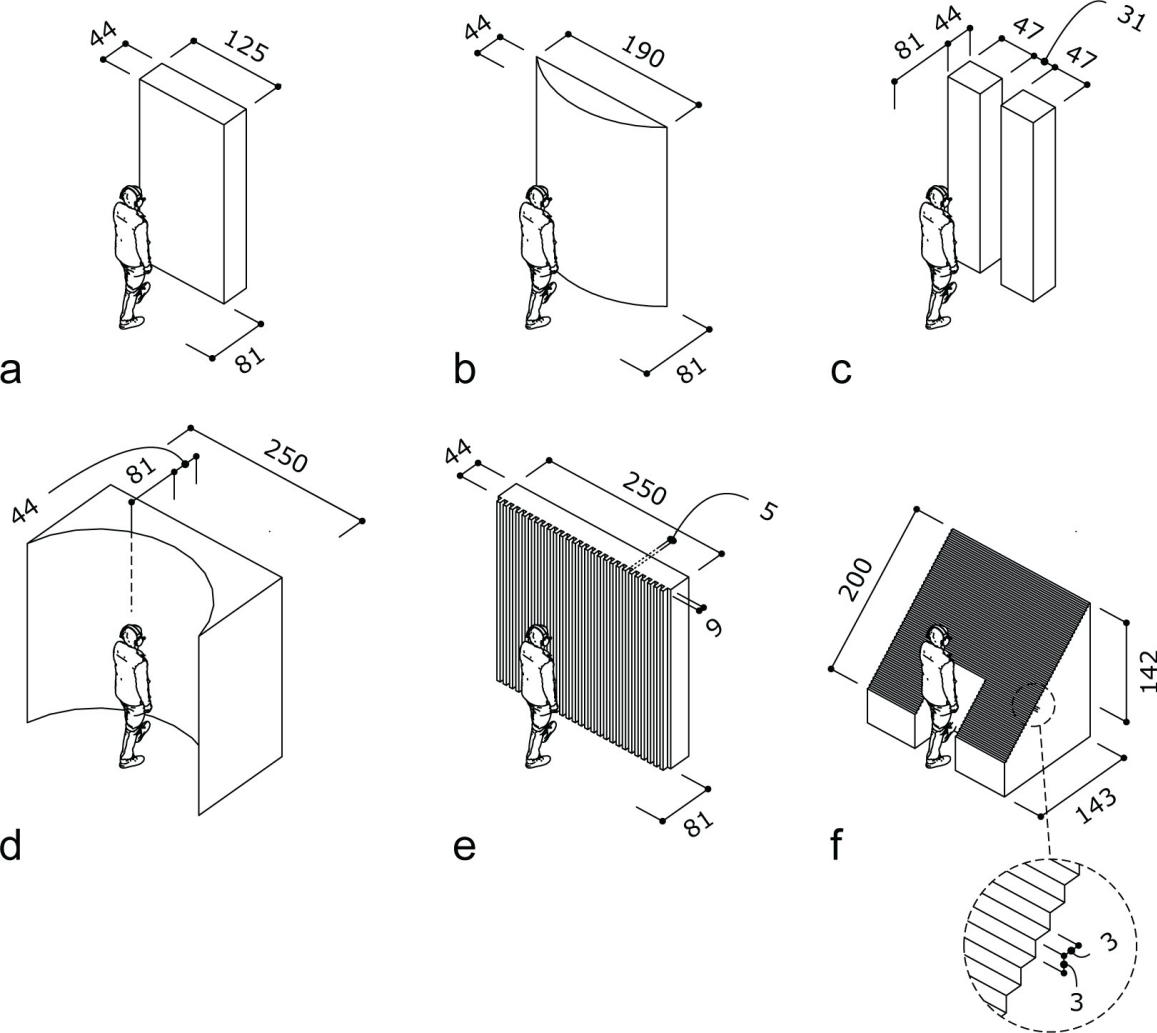
Drawings of the scenarios with nearby walls with different textures. The respective textures are a flat wall (a) a circular wall (b), a wall with aperture (c), a parabolic wall (d), a crenelated wall (e) and a staircase (f). The source is labelled as S. Dimensions are expressed in cm.

**Fig 4:**
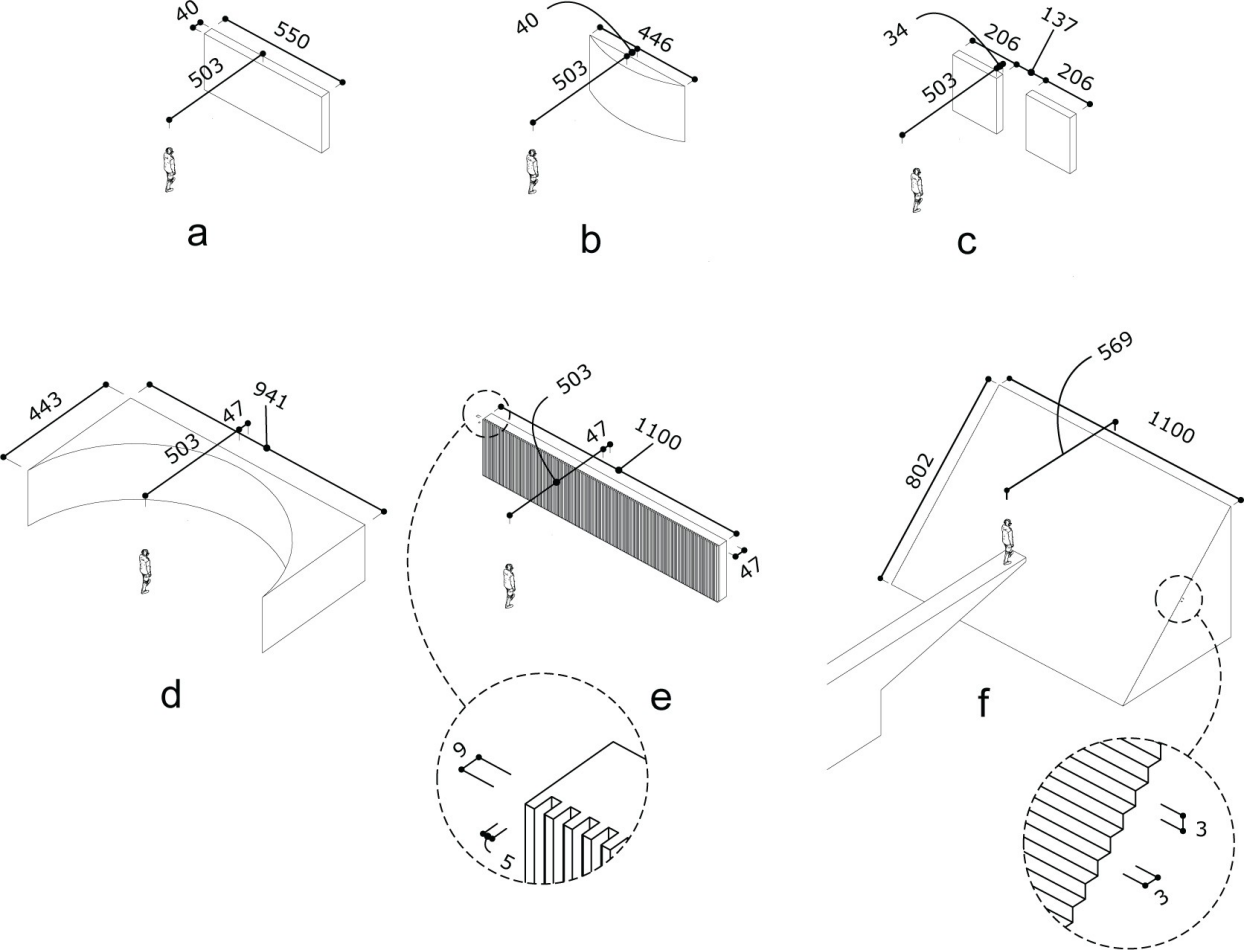
Drawings of the scenarios with far-away walls (5.0m) with different textures. The respective textures are a flat wall (a) a circular wall (b), a wall with aperture (c), a parabolic wall (d), a crenelated wall (e) and a staircase (f). The source is labelled as S. Dimensions are expressed in cm.

The study focusses on the investigation of 6 wall textures presented in Figs 3 and 4: a broad wall (a), circular convex wall (b), a wall with an aperture (c), a concave parabolic wall (d), a crenelated (periodic squared wave shaped) wall (e) and a staircase (f). The idea behind these choices is that these textures are already in use in buildings or they are feasible to construct in practice. Moreover, several of them are expected to present peculiar acoustic behavior (see Claes et al. [36] for spatial pattern of reflected sound waves) compared to a simple, flat wall (a), such as focusing (b and d), frequency dependent reflection (c), and frequency dependent angular dispersion (e and f).

Unlike classical echolocation tests, in which participants produce their own clicks in front of real objects, here we made use of a single (and thus perfectly reproducible), anechoically recorded click sound, which was convolved with an artificially made impulse response that corresponded with the transfer function between a point-like omnidirectional source and an omnidirectional microphone in an anechoic room containing a textured wall of interest. The simulated sound does not incorporate the directivity of clicks [15, 37], reducing somewhat the ecological validity of the results.

All walls were modelled as 100% reflective. The boundaries of the computation domain were simulated rigid. There was no need to model them as perfectly matching layers as the associated reflections were isolated from the impulse responses by time windowing of 6.0ms in case of the virtual wall or staircase nearby the person (at about 81cm) and 31.3ms in case of the virtual wall or staircase far away from the person (at about 5.0m). These two window lengthscorrespond to sound propagation distances of 2.05m and 10.72m, respectively. The sampling frequency was 192kHz, and the 2D grid resolution was 1.3mm. The plane of calculation was horizontal for all cases except the staircase, where it was vertical, due to the staircase being vertically textured. The receiver was placed a few cm above the transmitter. The simulation procedure is described in more detail in Claes et al. [36].

**Fig 5:**
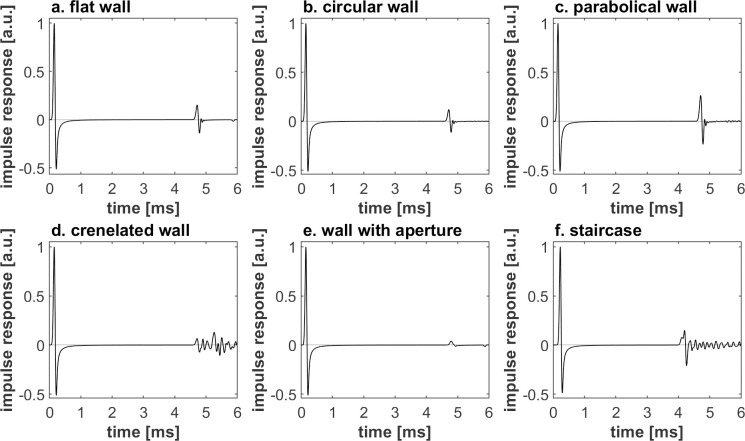
Normalized impulse responses of nearby walls (0.8m) with the following textures.

**Fig 6:**
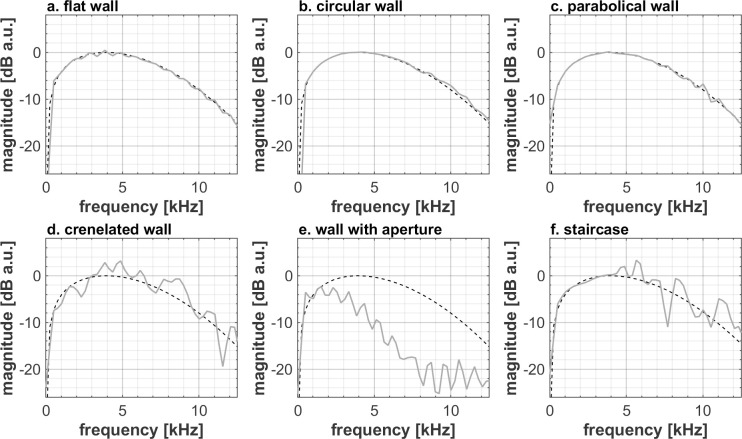
Spectral difference between the direct and reflected components (unconvolved) for each texture at 0.8 m distance. The spectra were obtained by Fourier transforming the two components without windowing, starting 0.05ms before the maximum of the direct sound and 3.85ms later for the reflected sound. The black and light grey curves respectively correspond to the direct and reflected components of the different binaural impulse responses. The spectra were normalized at 1.5kHz for the sake of better visualizing the coloration difference.

**Fig 7:**
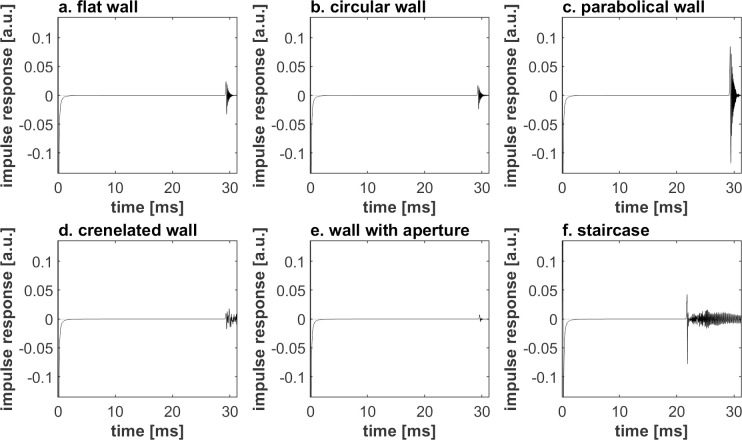
Normalized impulse responses of far-away walls (5.0m) with the following textures.

The stimuli of the different studied textures differ significantly with respect to their temporal structure and spectral coloration. At the short distance, early reflections of the flat (Fig 5A), circular (Fig 5B) and parabolic (Fig 5C) walls are impulsive. The t coloration caused by interference between the reflection and the direct component is not easy to interpret (Fig 6A–6C). In contrast, the early reflections of the crenelated wall (Fig 5D) and the staircase (Fig 5F) feature a long tail. For the crenelated wall, the tail is characterized by irregular oscillations. In the case of the staircase, the tail starts with a pulse, followed by oscillations corresponding to a series of reflections of increasingly distant steps. Similar envelopes of the reflected sounds can be seen at larger distances (Fig 7), albeit with increased complexity, later arrival and lower amplitude.

**Fig 8:**
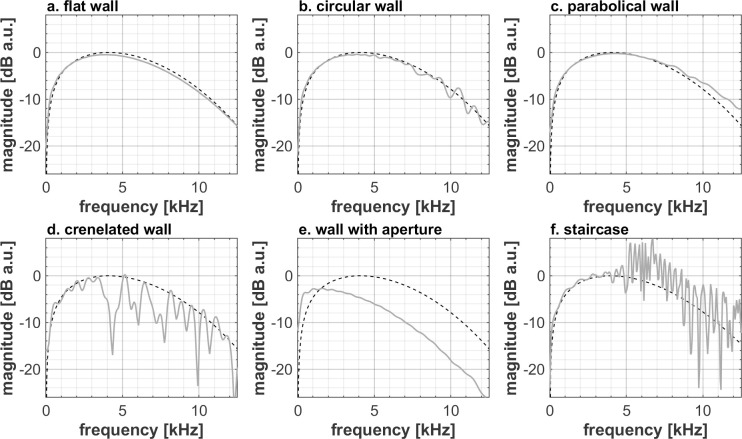
Spectral difference between the direct and reflected components (unconvolved) for each texture at 5.0 m distance. The spectra were obtained as described in the caption of Fig 6 except for the reflected sound, starting 20.6ms after the peak of the direct sound. The black and light grey curves respectively correspond to the direct and reflected components of the different binaural impulse responses. The spectra were normalized at 1.5kHz for the sake of better visualizing the coloration difference.

In addition to the loudness of an echo and its temporal features, provided the echo is long and strong enough to be audible and not masked by the direct sound, people’s ability to discriminate wall textures can also be based on differences in spectral content. Figs 6 and 8 show that after normalization, except for some small ripple or trend change (<1dB for 0.8m distance, <4dB for 5.0m distance) the spectra of a flat, a circular and a parabolic wall are quite similar to the ones of the direct sound, making it challenging to distinguish them on the basis of the spectrum. Compared to the direct sound and to the other wall types, the reflection spectrum of the crenelated wall and the staircase exhibit substantial irregularities above 3.5kHz. Due to high frequency, short wavelength components of the sound easily leaking through the hole, the reflection spectrum of the wall with aperture is missing high frequencies, giving a straightforward feature to distinguish this spectrum from the ones of the other wall types. Note that all spectra show characteristic, texture-related features all across the shown frequency range between 0.5kHz and13kHz, which is covered well by the used tongue click spectrum (Fig 1).

For better illustration of the perception of the sound stimuli, semantic description of the room impulse responses provided by few listening subjects is reported in the following. The flat surface was described as “basic, short, natural”, the circular surface as "short, neutral”, the parabolical one as “full, rich, strong”, the crenelated obstacle as “soft, longer”, the wall with an aperture as “hollow, empty, weak” and the staircase as “bird, arrow".

### Participants

The listening test experiment was conducted on 14 sighted persons, eight women and six men, between 22 and 54 years old (Average = 30, Standard Deviation = 11, Median = 25). All of the participants were performing this kind of listening test for the first time and none of them was an expert in acoustics. The participants were informed beforehand on the general purpose of the study without any details on the exact auralized scenario they were listening to. They all reported having normal hearing, which was also confirmed by a pure tone audiometry test (250Hz to 8kHz) that was supervised by a researcher with a screening audiometer AS7 (Kampler_®_) just before the actual test. All 14 participants had a hearing threshold lower than 30dB Hearing Level (dBHL) over all tested frequencies. Every participant had given an oral informal consent to perform this experiment to be used within research context. Ethics approval was granted by the Social and Societal Ethics Committee of KU Leuven (SMEC). The participants were volunteers and were not compensated in any way for their effort.

### Listening test setup

The listening tests were conducted in a semi-anechoic chamber with a volume of 125 m^3^ (dimensions 5.4m x 5.4m x 4.2m), well insulated from the outside world (background noise with sound pressure level *L*_p,f_ < 0 dB for each third-octave band within the audible range). Sound stimuli were digitally broadcasted from a control room (next doors to the lab) by means of a HPS IV (Head Acoustics_®_) listening unit via a Scarlett 6i6 (Focusrite_®_) soundcard through SPDIF set to a sampling frequency of 48kHz and a resolution of 16 bits. The stimuli were presented by high-quality open headphones HA II.1 (Head Acoustics_®_). The participants were seated at an office desk located in the corner of the semi-anechoic room. A computer screen was placed on the desk to display the graphical interface of the test. The participants could trigger each stimulus (without limitation on the number of times within the provided time (see further)) and selected their answer using either a keyboard or a silent mouse (Logitech_®_ M220). In view of ease of manipulation and of comfort feeling, the participants were not blindfolded and no instructions were given to keep their eyes closed or open.

#### Room impulse response configurations

In addition to the configurations described above, two artificially modified scenarios were studied, in which the magnitude balance between the reflected wave packet and the direct sound was modified. In the first scenario, the Reflected-To-direct Level Difference (RDLD) [38] was enhanced by 6dB. Expectations were that making the reflection more audible compared to the direct sound would decrease the impact of forward masking and thus test persons would perform better at distinguishing walls with different reflecting properties [39]. For the sake of completeness, it can be mentioned that changing the RDLD also changes the distance perception of the reflecting object (cfr discussion on direct-to-reverberant ratio in Zahorik et al [40]). The direct sound wave had a significantly higher amplitude than the reflected components of the RIR. The time separation of the RIR components was 4.6 and 29.3ms for 1.6m and 10m travelling distance respectively, to be compared with the click duration of 3.7ms. The click duration was determined following the approach presented by Thaler [16], which consisted in the estimation of the span during which the envelop of the signal is higher than -26 dB (5%) of the peak value. The onset of the click was determined from the 0.4ms moving average of the signal envelope; its offset was extracted from a nonlinear least-squares fitting a decaying exponential (Optimization toolbox from Matlab®) to the decaying part of the signal envelope.

In the second artificial scenario, the direct sound was removed. On one hand, this modification could be expected to improve wall identification due to the reduction of the masking of the silent reflection by the louder direct sound. On the other hand, removing the direct sound also removes the possibility for the echolocator to compare the strength and spectrum of the reflected and the direct sound, thus leading to loss of information. Also, after removal of the direct sound, there is no possibility for informative coloration of the sound due to interference between the reflection of interest and the direct sound. In order to reduce the effect of the amplitude cue in the analysis, the reflection sounds were equalized to have the same loudness. As standard models of loudness, such as the ones of Zwicker and Fastl [41], have been designed for evaluating stationary sounds, we have used Boullet’s model of loudness [42] as a measure for equalizing the impulsive click echo sounds. The latter model is adapted to impulsive stimuli. Given that the source strength was kept constant across walls, by performing this equalization, the remaining cues were the envelope of the reflected sound and its spectrum. The amplitudes of these oral-binaural room impulse responses were then equalized to the same sound pressure level as in the original case. The loudness estimation was made using a free Matlab® toolbox [43], computing the overall impulsive loudness expressed in Sone for each of the textured walls at both distances without any modification. The wall impulse responses were then equalized to their maximal loudness by means of dichotomy.

#### Binaural room impulse response emulation

In the preparation of the stimuli to be presented to the test persons, the above described RIRs were split into their direct and reflection components, and down-sampled to 48kHz in order to be compatible with the sampling frequency of the signals to be convolved with. The direct component, corresponding to the emitted sound heard without any interference from the environment, was convolved with an Oral-to-Binaural Transfer function (OBTF). The OBTF was calculated with a Boundary Element Method using FastBEM® software [44]. The OBTF is the complex transfer function of the sound pressure between the reference point of the ear, i.e. at 1cm from the entrance of the blocked ear canal, and the one of the mouth, i.e. at 25mm in front of the mouth opening. It therefore accounts for the impact of a person’s head on the perception of a self-emitted sound. The OBTF was computed with a head model from the OpenHear database [45], simplified to feature an average node separation of 4.4mm. The source was modelled as a monopole centered between the lips. The sound pressure levels were computed frequency by frequency from 40Hz to 12kHz in steps of 40Hz using FastBEM®. The time signal of the OBTF was reconstructed at a sampling frequency of 24kHz by applying an inverse Fourier transform and then resampled at 48kHz in Matlab®.

The reflected component of the RIR was convolved with a Head-Related Transfer Function (HRTF) for an azimuthal and elevation steering angle of 0°. At this angle, the HRTF models the influence of a human head on the perception of a sound striking from the front, which is the most realistic scenario for person trying to identify the texture of a wall of interest. We did not investigate scenarios under different orientations of the head, as the respective signals could be expected to add information only on the location of a reflector, but not on its texture. The HRTF was extracted using the SOFA Matlab® and Octave® API from the FHK HRTFs database acquired with a KU100 (Neumann®) Dummy Head at 48kHz in an anechoic chamber. The acquisition procedure is described in [46].

The two convolved components were reassembled into an Oral Binaural Room Impulse Response (OBRIR) [47] and convolved with a tongue click that had been recorded in a semi-anechoic chamber from a trained echolocator at 44.1kHz with an omnidirectional microphone placed at 50cm of the mouth along the propagation axis. The signal was resampled to 48kHz. The tongue click had a duration of 8ms (corresponding to a sound propagation distance of 2.7m) and a peak frequency at 4.1kHz, in the range of typical palatal clicks, which have durations between 3 to 15ms and a peak frequency between 3 to 8kHz [23]. The amplification of the sound reproduction system was calibrated using an impulse response of the direct component convolved with both the OBTF and the excitation signal. The stimulus auralization system was set to produce a sound exposure level (SEL) of 51dB(A), corresponding to a peak value of sound pressure level of 85.5dB(A), using a sonometer type 2236 (Brüel & Kjær®) at the entrance of the closed ear canal. This stimulus level corresponds to a moderately loud click within echolocation context. The corresponding auralized click in an anechoic environment, i.e. only accounting for the propagation between the mouth and the ear of the echolocator, had most of its energy between 400Hz and 6kHz, with a peak around 2kHz.

### Task and procedure

#### Task

The audibility of differences in wall texture was tested using a 2 Alternative Forced Choice [48] method, known as ABX-type listening test, relying on pairwise comparison. The ABX procedure involves 3 individually triggerable sounds (each reflected from one of the above introduced textures: (a) a circular wall (b), a wall with aperture (c), a parabolic wall (d), a crenelated wall (e) and a staircase (f)) referred as A, B and X. A and B were always different, and X was the same as A or B. The participants were asked to find whether X was identical to A or B and they were forced to choose one of the two propositions. In case no difference between the stimuli A and B was audible, the participant was instructed to arbitrarily choose one or the other. A 2AFC psychoacoustic experiment has a guessing limit of 50%. The discrimination between the tested textures for a given combination is proportional to the answering accuracy above this threshold. For the sake of statistical reliability and repeatability, each combination of wall textures was presented in random order 3 times. The typical time for participants to perform a single listening test, each related to one of the 3 impulse response configurations, corresponding to 3 times 15 combinations of 6 stimuli at 2 distances was about 17 minutes (min = 8, max = 28, Standard Deviation = 5, Median = 15).

#### Procedure

When the experiment was introduced to the participants, they were informed that each stimulus corresponded to a sound heard by an echolocator self-producing a tongue click in front of an obstacle at an undefined distance. Other than instructions regarding the control of the test software and familiarization with the listening task, no information was provided to the participant. The listening test protocol was programmed in Matlab^®^.

The participants could only trigger one stimulus at a time and all three stimuli had to be listened at least once before the answer buttons were enabled. There was no limit on the number of times a stimulus could be trigger. However, a software-controlled time limit of 30 seconds was set for each comparison. A message was displayed 10 seconds prior to the limit, to inform the user of the time remaining. In case no answer was given within this time span, the software passed to the next wall comparison, and the answer was classified as incorrect. In practice, the answering time limit was only reached in 5‰ of the cases, so that the effect of the above classification on the global statistics was insignificant.

The three RIRs configurations were treated in individual listening sessions, each separated by a 5 minutes break. This pause was systematically offered to participants to maximize their concentration on the test and informally survey them on the perceived difficulty of the test. The order of presentation of the three RIR configurations between session, as well as the texture combinations and their distance to a virtual echolocator within each subtest, were randomly assigned by the listening test software.

The duration of the three sessions was strongly subject-dependent and ranged between 29 and 75 minutes (Average = 51, Standard Deviation = 14, Median = 48).

## Results and discussion

The results of the listening test sessions were analyzed using a three-way repeated-measures analysis of variance (ANOVA) full-factorial model using the IBM Statistics SPSS 26® software. The quantity of interest was the texture discrimination performance, quantified by the discrimination fraction (DF), which we defined as the fraction of the total number of comparisons for which a given texture or group of cases was answered correctly (i.e. X was associated by the listener with the correct member (A or B) of the presented (A, B) pair). The three considered factors of the ANOVA were the wall textures, the distance group, and the room impulse response configuration. A Bonferroni *post hoc* correction was applied to the pairwise comparisons. The three factors are discussed in the following sections. In addition, both partial eta-squared (*η*_*p*_*^2^*) and eta-squared (*η^2^*) [49, 50] are provided to assess the effect size, i.e. the substantive significance of an effect. *η*_*p*_*^2^* is the most commonly used size effect for ANOVA repeated measure design and directly provided by SPSS®. However, some research guidelines advise to mention it with its relative *η^2^* value [51, 52]. In the context of this article, the values of *η^2^* are low due to the number of considered factors and interactions but they do enable to compare effect sizes.

### Influence of distance between the echolocator and the (virtual) wall

**Fig 9:**
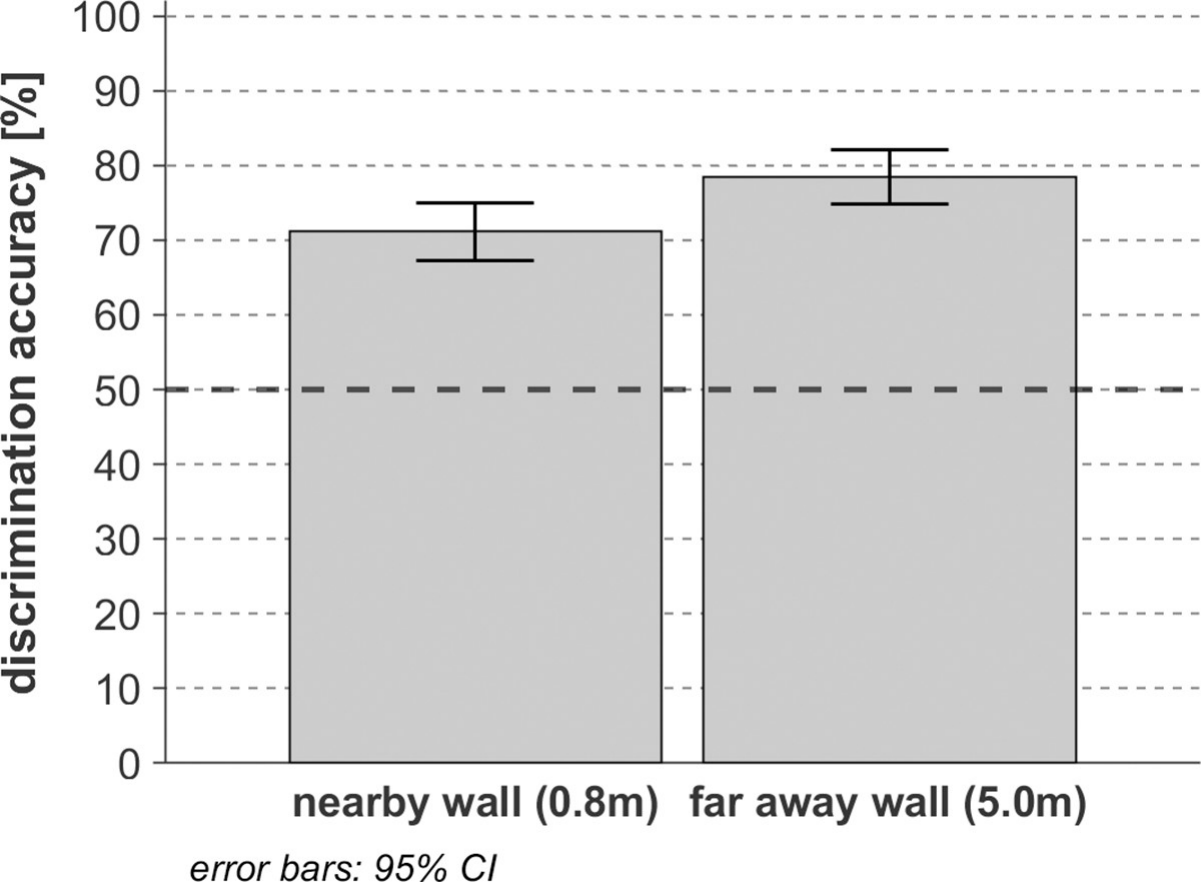
Texture discrimination performance grouped by distance as tested on 14 sighted participants. The level of accuracy corresponding to the guessing limit (50%) is represented by a dark black dashed line.

As shown in Fig 9, the average distinction score of the textures was significantly higher (*F*(1,13) = 26.09, *p* < 0.001, *η*_*p*_^*2*^ = 0.67, *η^2^ = 0*.*019*) for the walls located at 5 m distance (average DF = 0.79) than nearby, at 0.8 m distance (average DF = 0.71). This detection improvement may be related to the larger arrival time separation and thus smaller forward masking effect of the direct component of the OBRIRs on its reflected component at a longer distance. Apparently, this beneficial effect dominates the loss of RDLD in the long-distance case, in spite of this loss being substantial: between 4.5dB for the parabolic wall to 15.5dB for the crenelated wall. The loss of reflection magnitude with increasing distance would become harmful in cases where the reflection strength would drop below the hearing threshold or the background noise. Differences between closer walls being more difficult to distinguish than between far-away walls also indicate that the 5.7 and 6.5ms overlap (estimated based on the time interval of 95% (-26dBFS) of the central energy) between the direct and reflected click sounds in the case of close walls does not lead to information enhancing coloration but to masking-induced information scrambling.

It should be mentioned that, while the performed passive echolocation experiments have the advantage of featuring repeatable, good quality tongue click sounds, some of the conclusions made may not be extendable to a scenario in which an echolocator is performing active echolocation. It has been shown, that in the latter case, forward masking is much less of an issue [53].

For the sake of completeness, we note that the effect of a difference in distance between the echolocator and the wall is somewhat biased by the choice of the finite size of the considered walls. In time domain, the geometrical finiteness of the walls acts more or less as a temporal window: reflections from parts at different distances from the echolocator’s head arrive at different times, the latest reflections originating from the wall edges. In frequency domain, this window results in a convolution with the spectrum of the window function. Since the considered walls have the same size and since the convolution effect is quite limited at both distances (see e.g. the smoothness of the spectrum of the flat wall in Figs 6 and 8), it is safe to assume that the effect of the finite size on the results is very small. In any case, the walls at the shortest distance being discriminated worse than at the shortest distance indicates that reflections coming from large angles are not adding sufficient information to signal that would significantly help the recognition process.

### Effect of reflection magnitude enhancement

The investigation in the previous section allowed to assess the combined effect of a change in arrival time and amplitude of the wall reflection on the texture discrimination performance. In view of getting more insight in the recognition mechanism used for texture discrimination, in the following, we look into the separate effect of artificially enhancing the reflection magnitude, without changing the arrival time. We have investigated two artificial cases: one in which the direct sound was removed from the RIR, and one in which the reflection-to-direct ratio was increased by 6dB.

**Fig 10:**
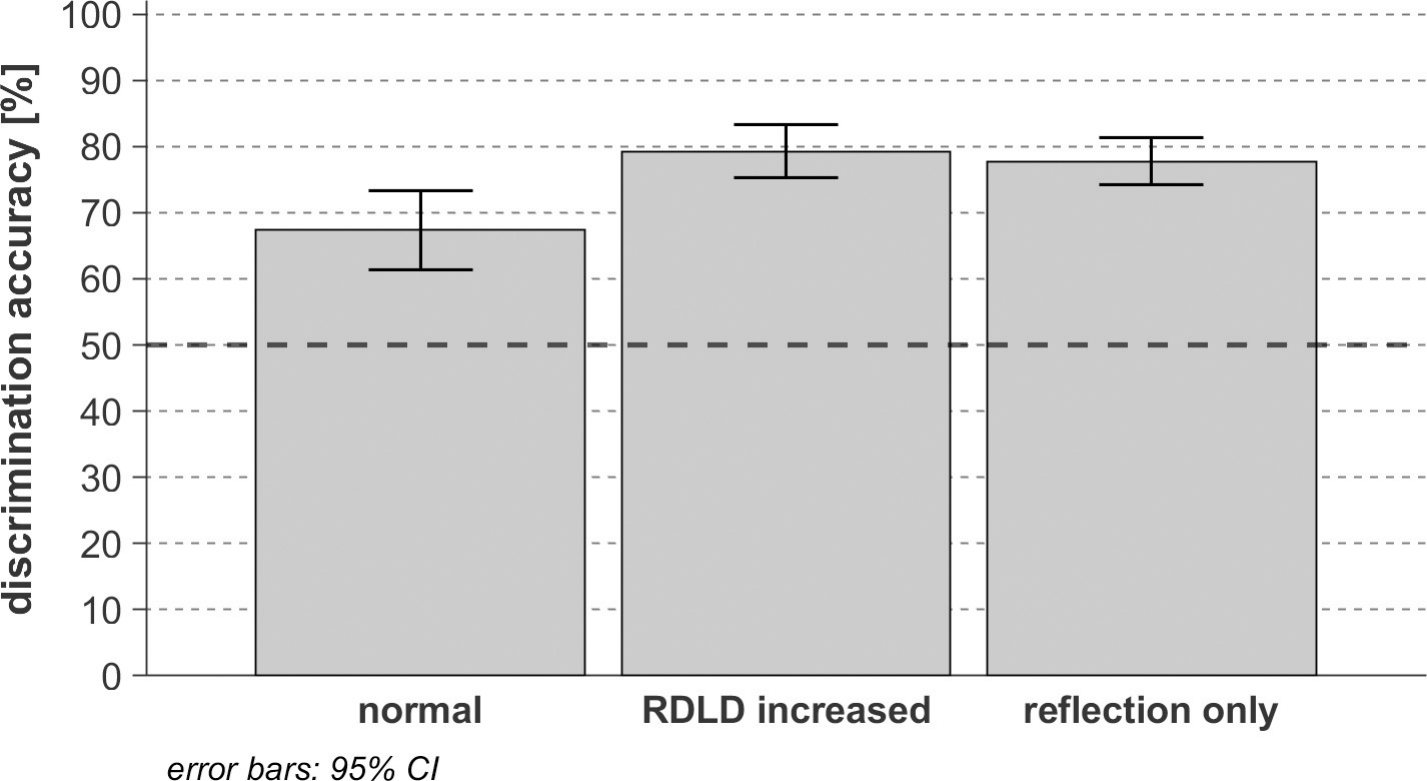
Texture discrimination performance for the three configurations of impulse responses tested on 14 sighted participants. The level of accuracy corresponding to the guessing limit (50%) is represented with a dark-black dashed line.

Fig 10 shows that overall, i.e. considering all wall textures and the two wall-person distances together, both modifications lead to a better discrimination performance (*F*(2,26) = 13.26, *p* <0.001, *η*_*p*_^*2*^ = 0.51, *η*^*2*^ = 0.039) compared to the natural case (average DF = 0.67, *p*_*Normal/RDLD increased*_ < 0.001, *p*_*Normal/Reflections only*_ = 0.02). This result confirms that for the considered configurations the direct component masks some meaningful information. However, fully removing the direct component of the convolved OBRIRs (average DF = 0.78) turns out not to lead to a significantly better texture discrimination than reducing its amplitude relative to the reflection (average DF = 0.79) (*p*_*RDLD increased /Reflections only*_ = 1.00).

**Fig 11:**
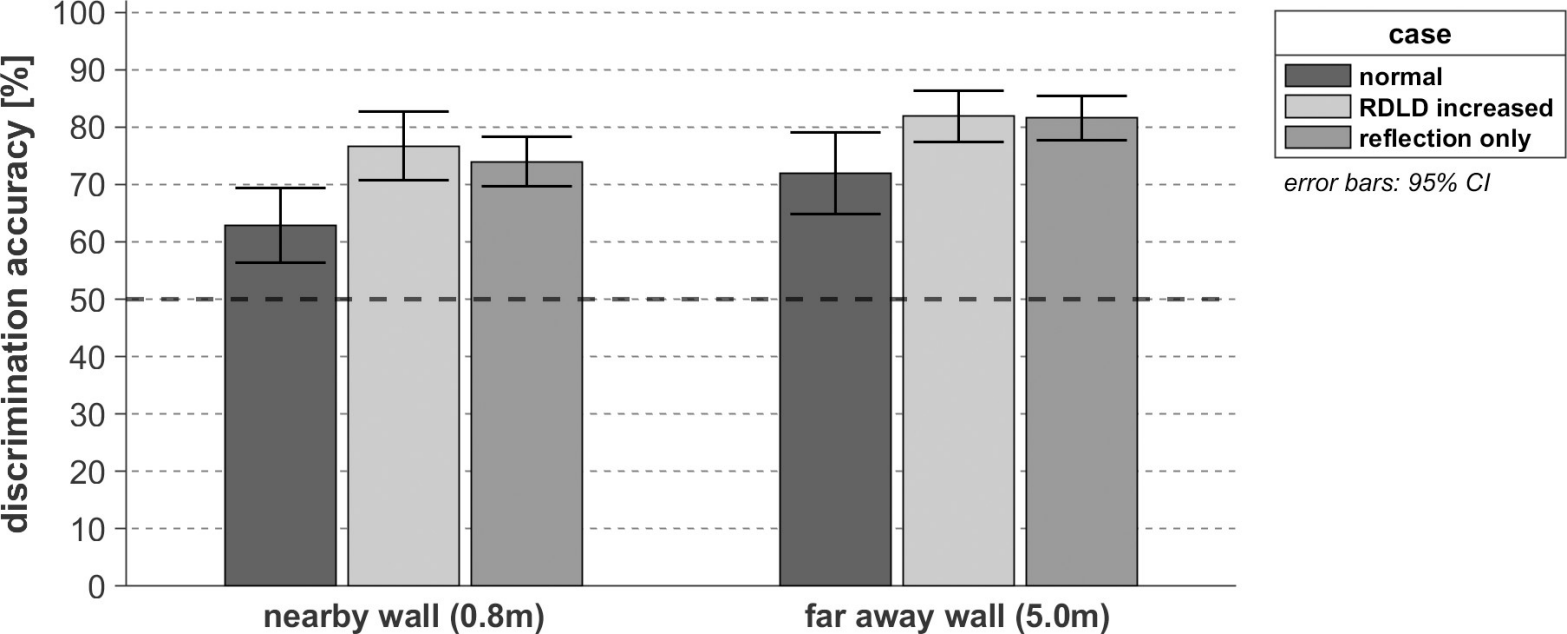
Texture discrimination performance for the interaction between the three configurations of room impulse response and the 2 distance groups investigated, tested on 14 sighted participants. The level of accuracy corresponding to the guessing limit (50%) is represented with a dark-black dashed line.

Interestingly, the latter observation does not only hold for the faraway walls (*p*_far_ = 1.00) but also holds for nearby cases (*p*_near_ = 0.95) as seen in Fig 11, showing that spectral coloration of enhanced reflections of a nearby wall caused by overlapping of the direct sound and reflection does not positively or negatively affect the wall discrimination performance. Previous research [19] found that both loudness and coloration cues are used to perform echolocation tasks at short distance (0.5m) by means of oral clicks, some participants reporting to rely on the tonal content of the reflections when the direct sound and reflections were overlapping, and therefore not perceived as separated sounds. This would suggest that the coloration cue in texture discrimination of close walls is faintly impacted by the coloration introduced by overlapping the direct and reflected components of the OBRIRs at 80cm distance. Furthermore, completely removing the direct component of the convolved OBRIR significantly improves the detection performance for nearby walls (*p*_near_ = 0.02) compared to the normal scenario. However, its effect on the discrimination between far away ones is too small to be significant (*p*_far_ = 0.07), indicating that the effect of masking in that case is limited, due to the substantial arrival time separation between the direct and reflected sound. The observation that removing the direct sound is clearly not deteriorating the discrimination performance but rather has a beneficial effect, indicates that listeners do not need information about the RDLD, and thus find sufficient information in the texture-dependent coloration of the reflected sound spectrum. However, it is important to keep in mind that the reflection level was enhanced in the absence of the direct component in order to match the sound pressure level observed when the direct sound was present. Therefore, the coloration cue might not provide such a clear detection enhancement at a non-enhanced reflection amplitude.

### Discrimination of different textures

**Fig 12:**
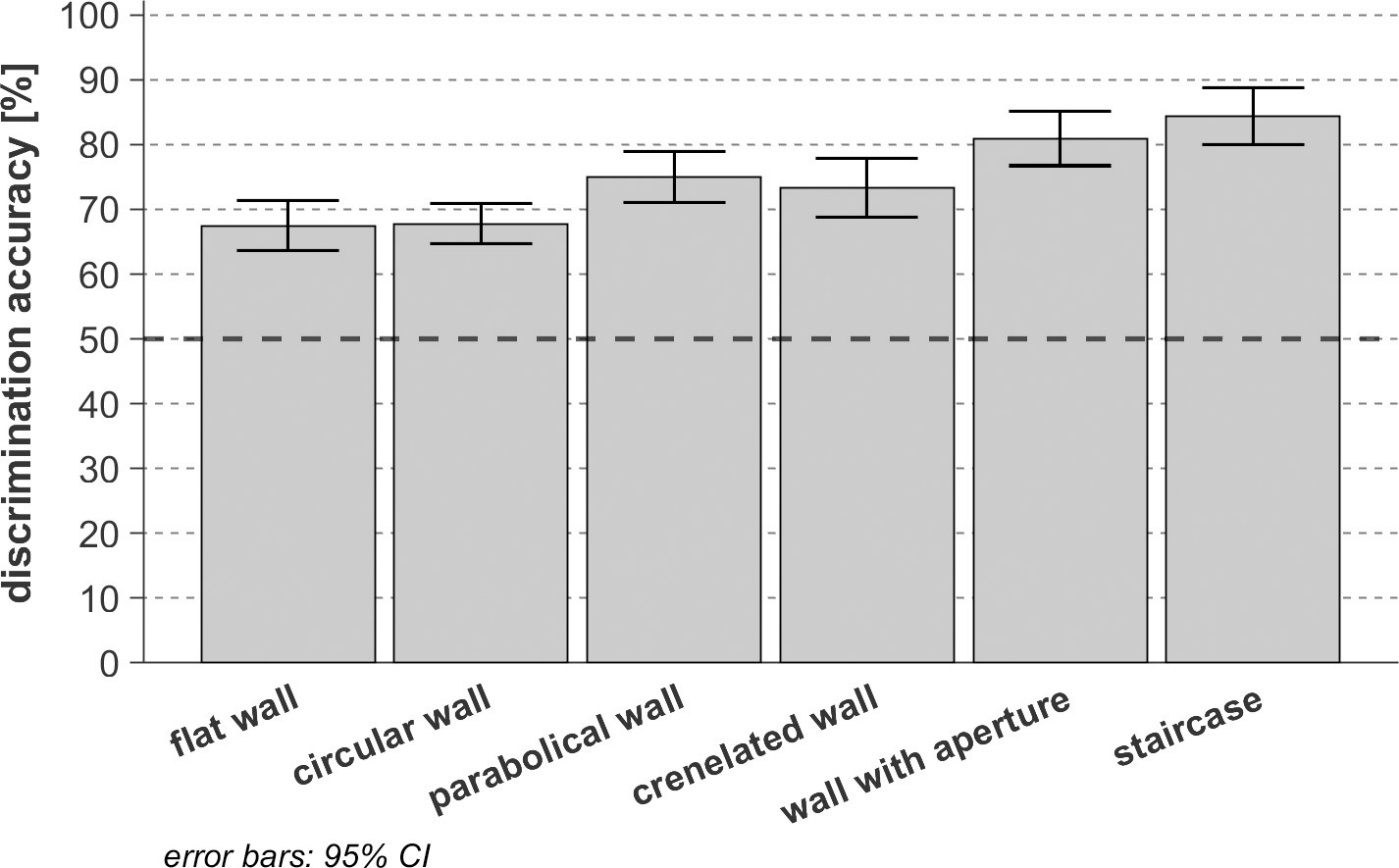
Overall texture discrimination performance tested on 14 sighted participants. The level of accuracy corresponding to the guessing limit (50%) is represented with a dark-black dashed line.

When analyzing the discrimination performance over the two distance conditions (0.8m and 5m) together, as shown in Fig 12, the textures could be clustered in 3 qualitative levels, each texture of a cluster being discriminated with a significantly different performance from these of the other cluster (*F*(5,65) = 40.47, *p* < 0.03, *η*_*p*_^*2*^ = 0.76, *η*^*2*^ = 0.055). Within each performance cluster, differences in discrimination performance between the cluster members are not significant (*p* > 0.91). The flat wall and the circular wall (first cluster) are the most difficult textures to discriminate, both averaging at 0.68 (i.e. 68%, only 18% above the 50% guessing limit). The parabolic and the crenelated wall (second cluster) are significantly easier to differentiate from other textures than the first cluster, respectively averaging at 0.75 and 0.73. The wall with aperture and the staircase (third cluster) are the most distinguishable textures, reaching detection rates of 0.81 and 0.84.

**Fig 13:**
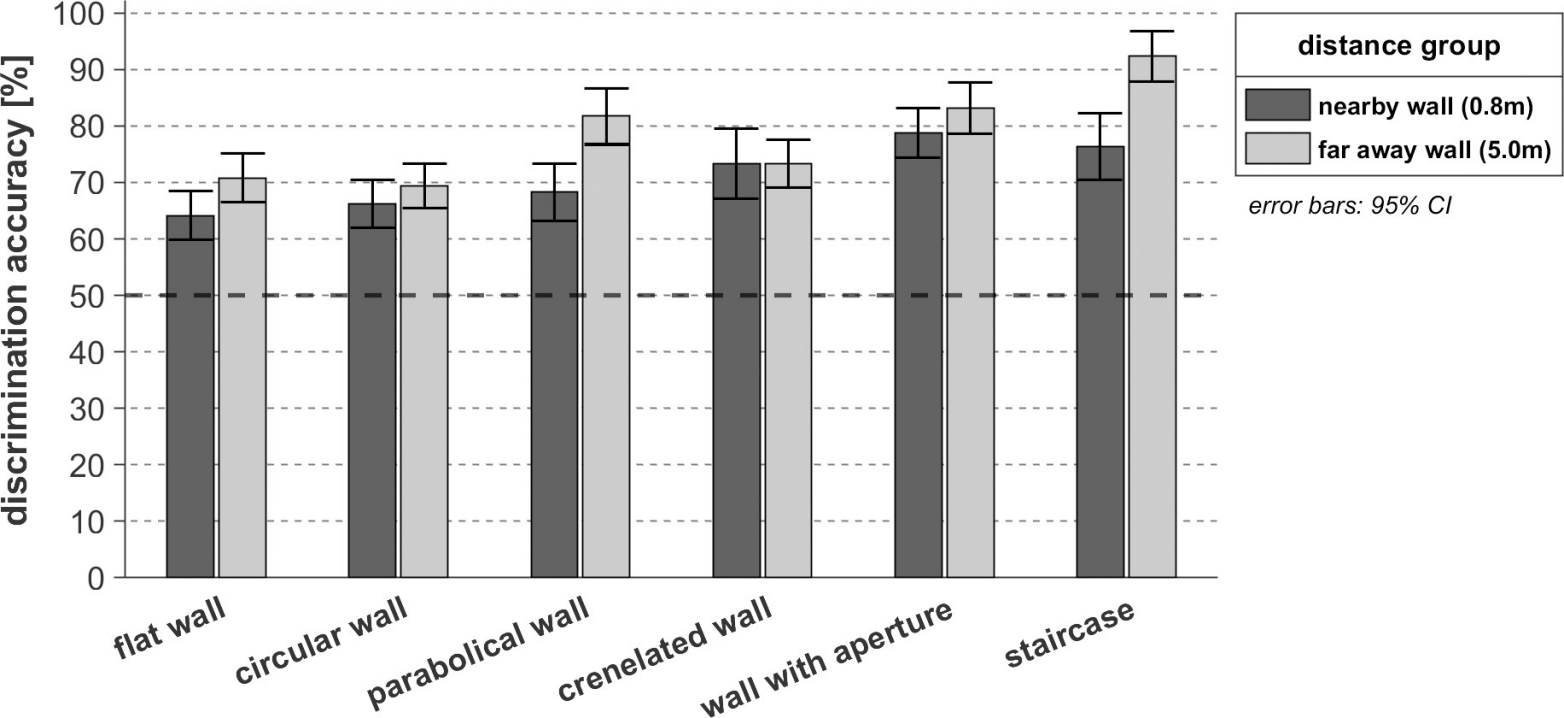
Audibility performance of the stated textures per distance group tested on 14 sighted participants. The level of accuracy corresponding to the guessing limit (50%) is represented with a dark-black dashed line.

Fig 13 shows that the above qualitative clustering is not adequate when looking at the two distance groups separately. However, some clear trends, different between the two distance groups, are salient. As discussed in the section related to the effect of wall distance over its discriminability, looking at all textures together, increasing the distance improves the discrimination performance. However, this trend was found to be very weak for the circular wall (*p* = 0.23) and absent for the crenelated (*p* = 1.00) walls. Due to its many ridges and resulting large number of non-specular, diffuse reflections reaching the observer at different times, the OBRIRs of the crenelated wall feature longer and less impulsive reflection tails than the other textures, except for the staircase. This suggests that the crenelated wall is less sensitive to the masking effect of the direct component and its overlapping on the reflections and therefore less prompt to discrimination improvement by increasing the wall distance. Furthermore, this indicates that the spectrotemporal features of the reflected sound from these wall types are not affected by masking by the direct sound. A discussion of the audibility of diffusion at different distances from a scattering surface is given in [27, 54, 55].

Looking at the impulse responses in Fig 5 in the previous section related to the stimuli, the reflected sound of the circular wall is characterized by an absence of spectral coloration and dispersion (distinguishing it from the reflections of the crenelated wall and the staircase shaped wall) and by the difference between its level and the one of the other wall types. The reflection magnitude of the circular wall is not so different from the one of a flat wall, explaining why the DF of flat wall-circular wall comparisons is poor (DF_flat_ = 0.68, DF_circular_ = 0.68, *p* = 1.00). Its reflection magnitude is also substantially higher than the one of a wall with aperture (> 7.5 dB) and substantially weaker (< -6.8 dB), due to the convex shape, diverging the reflected energy, than the one of a parabolic wall, which concentrates the reflected energy towards the listening location. This difference in reflection magnitude explains why the circular wall could be reasonably discriminated from those wall types (DF_circular_ = 0.68, DF_with an aperture_ = 0.81, DF_parabolic_ = 0.75, *p*_*circular/ with an aperture*_ < 0.001, *p*_*circular/ parabolic*_ < 0.001). The discrimination difference with the wall with an aperture occurs at both distances (*p* < 0.002), as opposed to the one with the parabolic wall, only occurring at the longer distance (*p* < 0.001).

**Fig 14:**
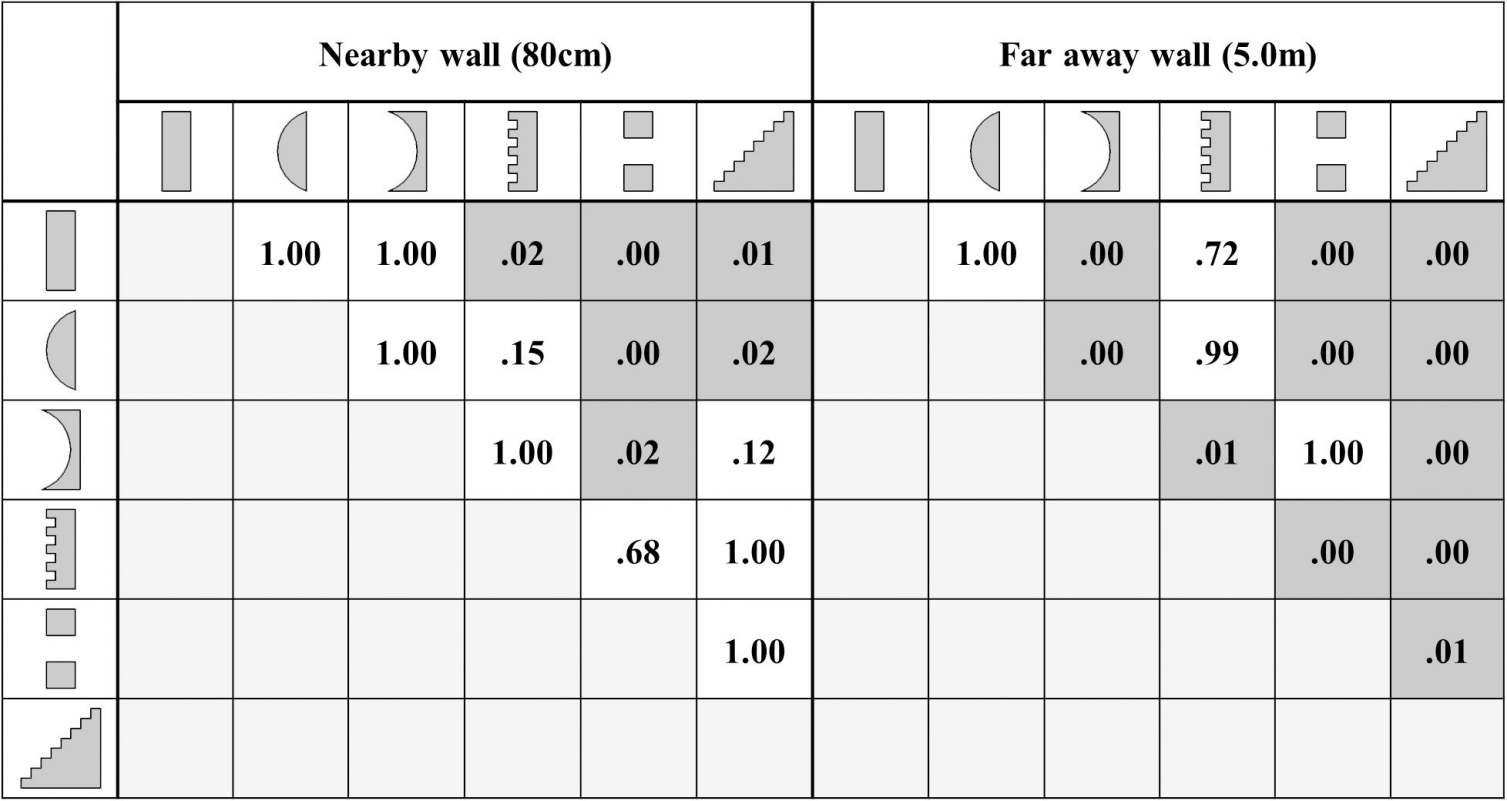
Listening test based statistical significance (p-value) of the discrimination fraction of the presented texture pairs at the two investigated distance. The right and left sections of the table respectively correspond to 0.8m distance and 5.0m distance. The pairs that are significantly discriminated are greyed out. The included drawings provide a schematic representation of the textures and depict neither the size nor the layout of the simulated ones.

A summary of the significances (p-value) of the discrimination between pairs of wall types is listed in Fig 14.

At 5 m distance, without masking effects, as a consequence of the clear dispersion resulting from the train of arrivals of partial reflections from the different steps, and the associated spectral coloration, the staircase-shaped wall is clearly the easiest to recognize (DF = 0.92, *p* < 0.01). At 0.8m, the discrimination performance for the staircase wall is lower (DF = 0.76), though still higher than the one of other wall textures, except for the wall with aperture (DF = 0.79). In general, the wall with aperture is also well distinguished from the other wall types, due to the lack of reflection by the aperture, in accordance with observations by Calleri et al. [33]. This results in a significantly weak reflection amplitude compared to the other wall types. Furthermore, there is a considerable reduction of the high frequency content of the reflection due to diffraction on the aperture. As a result, the discrimination based on texture coloration becomes more effective in the low frequency range [20]. The performance of distinguishing a wall with aperture from another wall is only poor with respect to a crenelated wall at 0.8m (DF _aperture/0.8m_ = 0.79, DF_crenelated/0.8m_ = 0.73, *p* = 0.68) and to a concave parabolic wall at 5 m (DF_aperture/5.0m_ = 0.83, DF_parabolic/5.0m_ = 0.82, *p* = 1.00).

**Fig 15:**
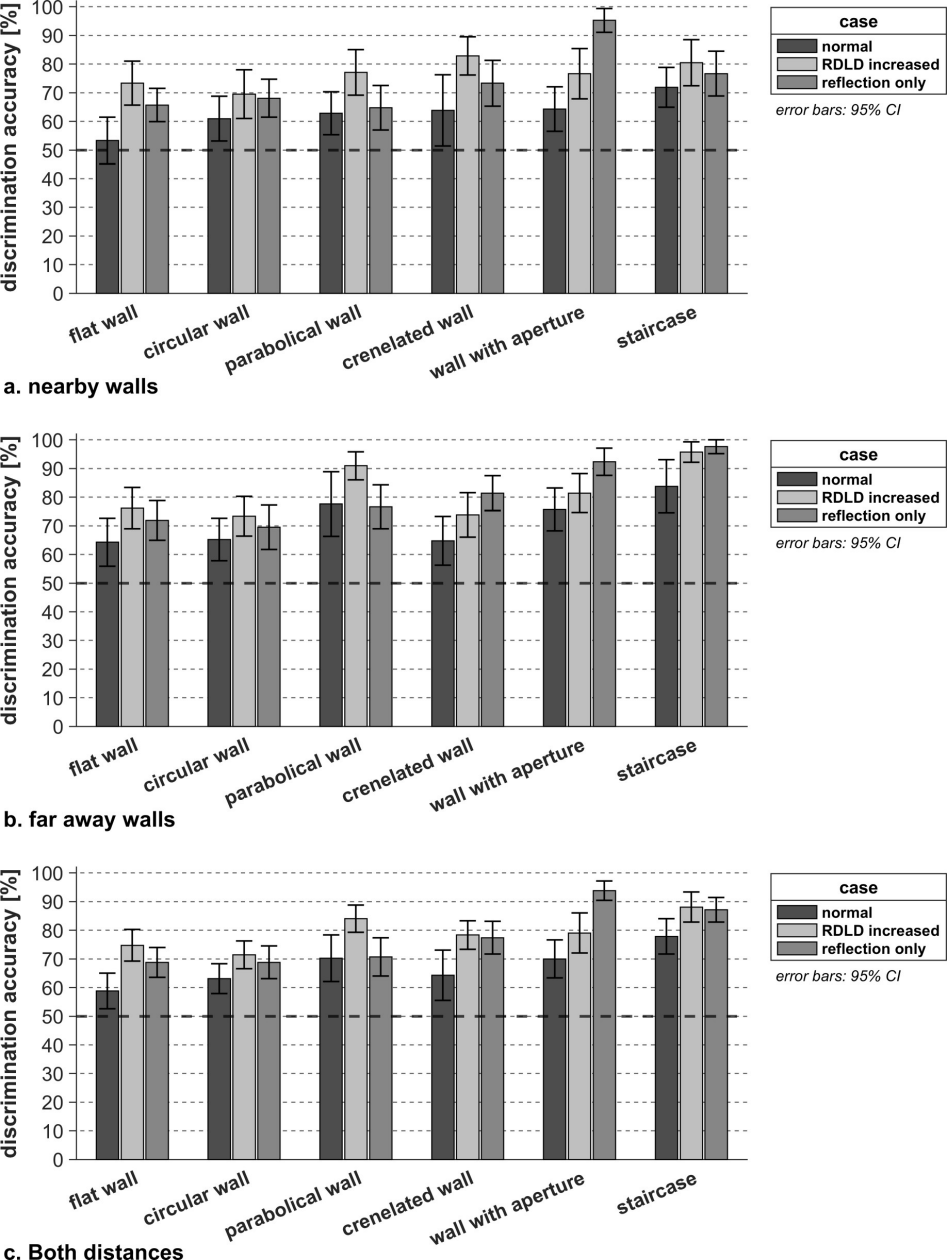
Texture discrimination performance per configuration of room impulse response tested on 14 sighted participants per distance group. This figure is split into 3 parts, respectively corresponding to the nearby walls (a.), the far away walls (b.) and textures at both distances (c.). The level of accuracy corresponding to the guessing limit (50%) is represented with a dark-black dashed line.

Fig 15C summarizes the effect of enhancing the RDLD or removing the direct sound on the DF for each wall type. Except for the convex circular wall, the DF increases (though not always significantly) with decreasing relative strength of the direct sound, confirming the earlier observation that masking by the direct sound is often deteriorating the clarity of information in the reflected sound. Except for the wall with aperture, statistically significant gains in DF by RDLD enhancement are found for all wall types (*p* = 0.08). Removing the direct component and equalizing the loudness of the reflections does not significantly improve the DF for the circular (*p* = 0.65) and the parabolic wall types (*p* = 1.00).

Further recognition enhancement when fully removing the direct sound rather than just making the RDLD stronger is only significant for the wall with an aperture (*p* <0.01). This stresses the importance of the reflection coloration for the latter wall type outperforming in the absence of its direct sound, i.e. without any information on the reflection arrival time and direct RDLD level comparison. The concave parabolic wall is easier to differentiate from other wall types when the RDLD is increased than when the direct sound is removed and the sounds to compare are equalized (*p* = 0.01).

### Design and analysis of single quantities number for texture discrimination

**Fig 16:**
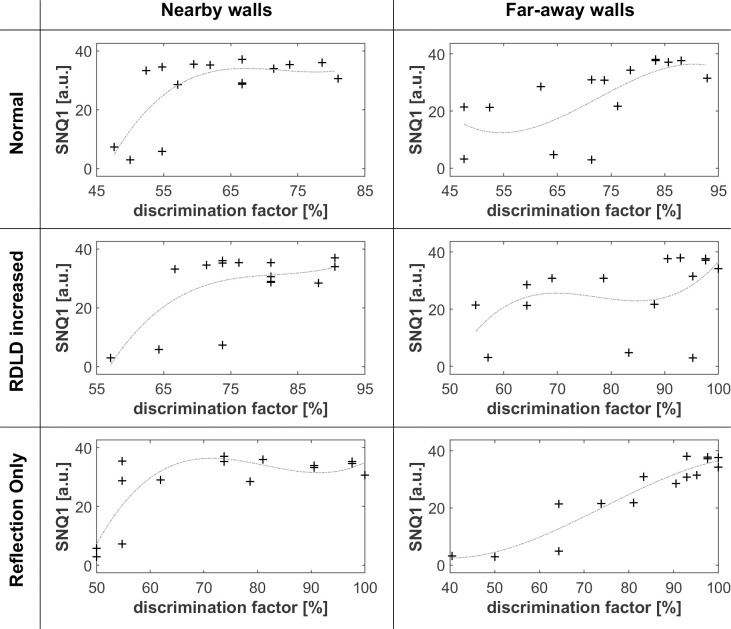
Correlation between the difference between time signals of texture pairs (SNQ1) and discrimination performance. SNQ1 consists in the rms value of the difference between the time signals of each pair of textures. The dashed light grey curves are 3rd order polynomial fits of the rms difference between the time signals with the related discrimination factors.

**Fig 17:**
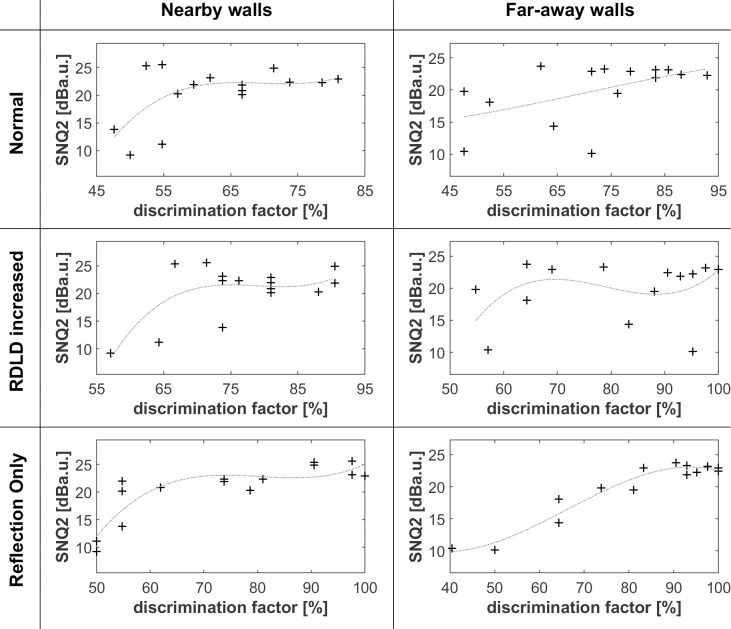
Correlation between the difference between spectra of texture pairs (SNQ2) and discrimination performance. SNQ2 consists in the rms value of the difference between the frequency domain of each pair of textures. The dashed light grey curves are 3rd order polynomial fits of the rms difference between the time signals with the related discrimination factors.

**Fig 18:**
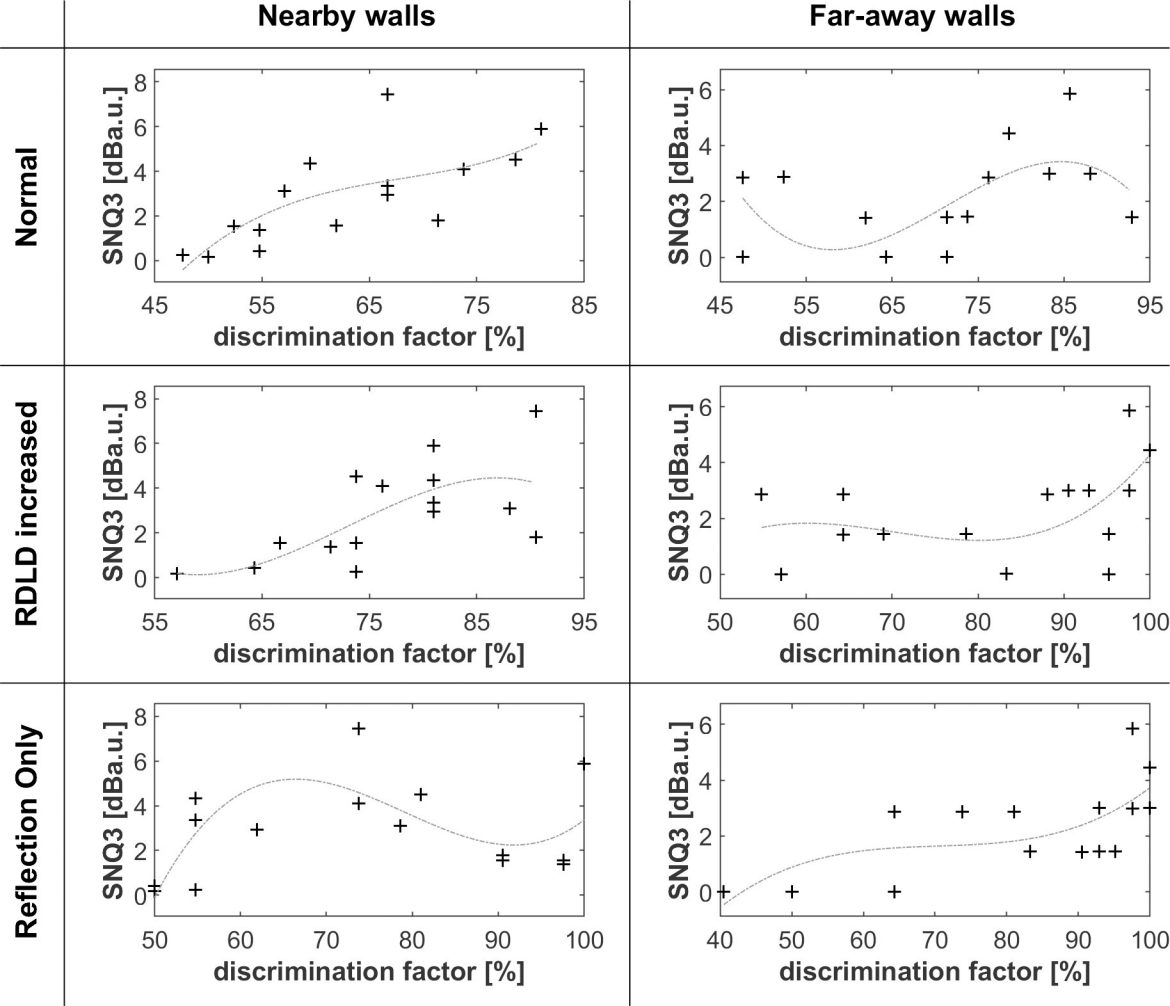
Correlation between the difference of non-smoothness spectra of texture pairs (SNQ3) and discrimination performance. SNQ3 consists in the absolute value of the difference between the rms values of the numerically differentiated spectra of each pair of textures. The dashed light grey curves are 3rd order polynomial fits of the rms difference between the time signals with the related discrimination factors.

Figs 5–8 shows that differences in wall texture are causing clear differences both in echo time signal features and in spectral features (the shown spectra are for the echo only, after removing the direct sound). It could be expected that the discrimination performance of people for a certain wall pair is strongly related with the degree of difference in spectral and/or temporal features. In order to investigate this, we have plotted in Figs 16–18 the correlation between three single number quantities (SNQ) that represent respective spectral or temporal differences between the two members of all 15 wall pairs, i.e. each possible pairing of the 6 textures, and the average discrimination performance for those wall pairs at each distance.

SNQ1 (Fig 16) is the rms value of the difference between the time signals. SNQ2 (Fig 17) is the rms value of the difference between the spectra. SNQ3 (Fig 18) is the absolute value of the difference between the rms values of the numerically differentiated spectra. This quantity reflects the difference in non-smoothness of the spectra. A visual inspection of the spectra shows that different walls have a clearly different degree of non-smoothness in their spectra.

Fig 16 shows a mixed picture, with, depending on the combination of wall distance (far/nearby) and stimulus presented to the listening people (normal, RDLD enhanced, echo only), a faint or poor correlation between time signal related SNQ1 and people’s DF. There seems to be some correlation for a normal echo and RDLD increased stimulus (where the direct sound might act as a kind of reference), not for reflection only stimuli.

Fig 17 shows some degree of correlation in the case of a far wall and reflection only, suggesting that when the echo spectra are not masked by the direct sound, their difference can to some extent be detected.

Fig 18 shows some degree of correlation in the case of a nearby wall with a normal or RDLD enhanced echo. Apparently, people’s perception of the non-smoothness of the reflection spectrum is enhanced when the echo can be referenced with the direct sound.

Overall, the found correlations are relatively weak, and it can be expected that a test person is able to take multiple spectro-temporal features into account when trying to decide whether two echoes are different or not. In view of that, we have also verified to what extent the *combination* of the three discussed SNQs is correlated with people’s discrimination performance. We have tackled this question by training an artificial neural network (ANN) to predict, starting from the {SNQ 1, SNQ2, SNQ3} values of the 15 wall pair combinations as training examples, the average discrimination performance as found from 15 (wall i, wall j)-pair-comparisons during the listening tests. The discrimination performances found from the 15 reverse order (wall j, wall i)-pair-comparisons were kept as test examples. The neural network had 3 SNQ inputs and one bias, 2 hyperbolic tangent input neurons, and a linear output neuron. The iterative ANN-training was stopped just before the test error started to increase, in order to minimize overtraining effects.

**Fig 19:**
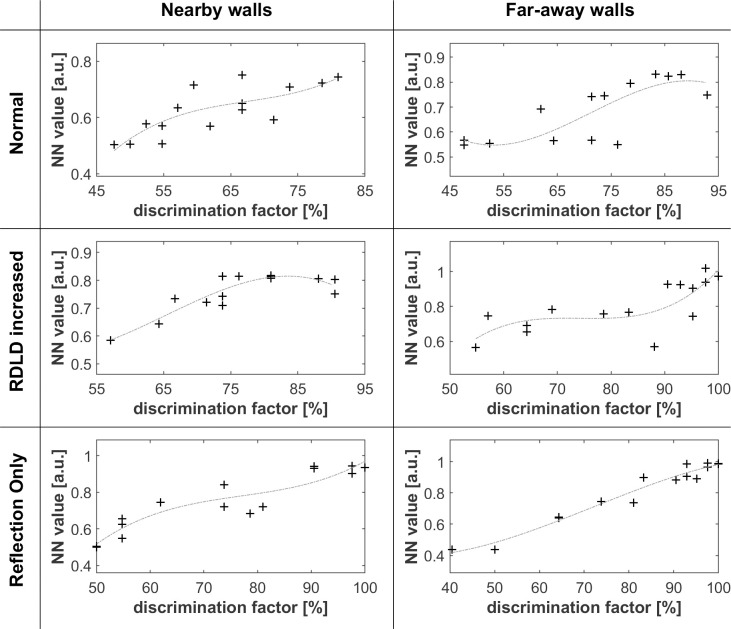
Correlation between the ANN-predicted discrimination performance and the discrimination performance that came out from the listening tests. The dashed light grey curves are 3rd order polynomial fits of the rms difference between the time signals with the related discrimination factors. Plus symbols are for the 15 training data, circles are for the 15 test data.

Fig 19 shows the neural network training and test performance for predicting the DF based on normal echo, RDLD enhanced echo and echo alone, for far away wall pairs. Except for a few test example outliers, the rather simple (only 2 hidden neurons) ANN function predicts quite well the DF, indicating that the combination of the 3 SNQ’s, signal pairs has a similar degree of information content as the spectrotemporal features that are used in the subjective discrimination process by the average listening test person.

## Conclusion and perspectives

By using a passive echolocation paradigm, for which the excitation source is a tongue click virtually located at the mouth position, on average, 14 sighted participants with no prior experience in echolocation tasks were moderately successful in discriminating most of the investigated wall textures from the others. Different textures could correspond to different “messages”. This indicates that, provided training, textured architectural elements in spaces could be used to assist visually impaired people to orient themselves by interpreting cues in reflections of self-made sounds.

### Distance

The discrimination performance was higher at the longest distance (5.0m), suggesting that the direct sounds mask some meaningful information of the reflections at the shortest distance (0.8m) thereby reducing the discrimination performance at that distance. The investigation of intermediate distances would be profitable to assess the distance dependence of the discrimination performance of these textures. Setting the wall distances to 2.8 and 3.6m, respectively corresponding to a separation time of the 2 components by 5 and 10ms (estimated based on the time interval of 95% (-26dBFS) of the central energy) using identical transfer functions and excitation signal, would be interesting to assess how the texture discrimination performance is influenced by the wall distance for a significant lag.

### Excitation stimulus

The tongue click used in this experiment is impulsive (duration = 3.7ms with a threshold criterion set to -26dBFS, i.e. 5% of the total energy) but includes a low amplitude tail (duration 8.5ms with a threshold set to -32dBFS, corresponding to 2.5% of the energy), which tends to reduce the arrival times between the direct and the reflected sounds and therefore increases the masking effect of the direct sound. Echolocation experts are able to generate even shorter palatal clicks [15], which should improve the discrimination performance of the texture at short distance. Other excitations such as hisses are used for short distance detection relying on coloration discrimination [19] and not on the perception of 2 separated events. Considering that the investigated textures have strong coloration for the walls featuring a non-continuously flat surface, the use of hisses should be beneficial in this context.

### Room impulse response configurations

The modification made on the IRs to either enhance the reflection amplitude by 6dB or to remove the direct component–though equalizing the loudness of the reflections–both improve the discrimination performance of the wall textures compared to a normal echo cue.

This is interesting with respect to the development of training tools using a real-time convolution server, suggesting that the coloration detection could be specifically trained by artificially removing the direct component of the IRs fed to the convolution engine. This specific coloration training would however require the use of a closed-back headphone to limit the perceived energy of the airborne propagation of the excitation noise when self-producing a sound. The direct component of the excitation will however still be perceived by the echolocator through bone conduction with a significantly reduced magnitude. The difficulty of an echolocation task in such a training tool could be set by tuning the RDLD value. Beginners could start with a substantial amplification (between 6 and 10dB for example) of the reflected component of the IRs and decrease it over time. This modification would not require closed-back headphones.

Another test could be to artificially increase the separation time between the direct and reflected components of the BRIRs, only considering this parameter in the investigation. However, we could assume that texture discrimination would decrease with distance [1, 56] from the location where the direct sound no longer masks the reflections.

### Spectral coloration

The reflection coloration of the IRs was proven to be the main cue for texture recognition. Discrimination by echolocators between architectural markers placed in conventional spaces, i.e. rooms or corridors created by an assembly of flat walls, would thus be favored if they induced a strong coloration of the reflection and if the reflection were strong enough to limit the masking effect of the other signal components, such as the direct sound or the background noise.

### Limitation of the realism of the auralizations

#### 2D scenario

In this work, we have looked at the discrimination performance using RIRs computed by a 2D finite difference method with a point source which corresponds to a line source in a 3D space, therefore increasing the amount of reflection bounced back to the echolocator. This might have improved the discrimination fraction compared to a point source in a 3D room representative of an echolocation task. An extension of this work to 3D could shed light on this.

#### Head movement

The different walls in this article were perceived at a fixed azimuthal angle. Head movements have been shown to be highly beneficial for echolocation tasks [5], especially in complex environments [57]. The use of multiple head orientations and therefore multiple angles of incidence and reflection of the click sound on the wall, would provide additional spatial information on the obstacles, and can be expected to improve people’s recognition performance.

#### Texture walls in an anechoic environment

In this study, the detectability of texture differences in a space with a realistic reverberation time has not been tackled. Time windowing the IRs, and thus discarding reverberant sound, might have had two opposite effects. On one hand, discarding the late part of long reflection tails of some might have affected their information content. On the other hand, limiting the impulse response length removes information scrambling reflections of objects/walls other than the one of interest [58, 59]. However, late reflections of a room with a moderate reverberation time might sometimes be beneficial to improve wall detection at large distances [18] (TR = 0.4s) and has been shown to improve detection performance of obstacles through echolocation [59] (TR = 1.4s). For future work, it would be interesting to verify the effect on the texture discrimination performance of taking into account the reverberation tail of a real-life room. We are somehow confident that several of the conclusions made here for simulated sounds, especially those concerning which wall pairs can be most reliably discriminated, will still hold in real life circumstances: sound source localization experiments, in which the performance also depended on spectro-temporal cues, have shown good correlation of the localization performance between sound received by own ears and auralized sound [60].

#### A passive investigation of an active phenomenon

The discrimination performance with self-made tongue clicks in active echolocation experiments can be expected to follow the same trends as the ones observed here with passive echolocation, with some variability related to the spectral content of self-made clicks [15, 39] and to forward masking of information on texture in reflections of self-made clicks being less harmful than with recorded clicks [53].

## Supporting information

S1 File Discrimination factor of all 14 participants for each studied pairwise comparison at the two considered distances (0.81 and 5.03m) and for all three RDLD configurations. Each participant is referred as an arbitrary index. 10.6084/m9.figshare.13553582.
(XLSX)

## References

[1] Rowan D, Papadopoulos T, Edwards D, Holmes H, Hollingdale A, Evans L, et al. Identification of the lateral position of a virtual object based on echoes by humans. Hear Res. 2013;300: 56–65. 10.1016/j.heares.2013.03.005 23538130

[2] Kolarik AJ, Cirstea S, Pardhan S, Moore BCJ. A summary of research investigating echolocation abilities of blind and sighted humans. Hear Res. 2014;310: 60–68. 10.1016/j.heares.2014.01.010 24524865

[3] Griffin DR. Echolocation by blind men, bats and radar. Science (80-). 1944;100: 589–590. 10.1126/science.100.2609.589 17776129

[4] Fletcher NH. Animal bioacoustics. Springer Handbook of Acoustics. Springer; 2014. pp. 821–841.

[5] Milne JL, Goodale MA, Thaler L. The role of head movements in the discrimination of 2-D shape by blind echolocation experts. Attention, Perception, Psychophys. 2014;76: 1828–1837. 10.3758/s13414-014-0695-2 24874262

[6] Thaler L. Echolocation may have real-life advantages for blind people: An analysis of survey data. Front Physiol. 2013;4: 98. 10.3389/fphys.2013.00098 23658546 PMC3647143

[7] Devlieger P, Frank R, Froyen H, Wildiers K, editors. Blindness and the multi-sensorial city. Antwerpen: Garant; 2006.

[8] Dodsworth C, Norman LJ, Thaler L. Navigation and perception of spatial layout in virtual echo-acoustic space. Cognition. 2020;197: 104185. 10.1016/j.cognition.2020.104185 31951856 PMC7033557

[9] Kish D. Human echolocation: How to “see” like a bat. New Sci. 2009;202: 31–33.

[10] Gay J, Umfahrer M, Theil A, Buchweitz L, Lindell E, Guo L, et al. Keep Your Distance: A Playful Haptic Navigation Wearable for Individuals with Deafblindness. ASSETS 2020 - 22nd Int ACM SIGACCESS Conf Comput Access. 2020; 8–10. 10.1145/3373625.3418048

[11] Garcia S, Petrini K, Rubin GS, Da Cruz L, Nardini M. Visual and non-visual navigation in blind patients with a retinal prosthesis. PLoS One. 2015;10: 1–12. 10.1371/journal.pone.0134369 26225762 PMC4520559

[12] Jicol C, Lloyd-Esenkaya T, Proulx MJ, Lange-Smith S, Scheller M, O’Neill E, et al. Efficiency of Sensory Substitution Devices Alone and in Combination With Self-Motion for Spatial Navigation in Sighted and Visually Impaired. Front Psychol. 2020;11. 10.3389/fpsyg.2020.01443 32754082 PMC7381305

[13] Dujardin M. End-user driven architectural studies in Belgium through student participation in real multi-stakeholder projects and the development of a training programme for user-experts. rd Int Conf Univers Des Hamamatsu, Japan. 2010;2.

[14] Rojas JAM, Hermosilla JA, Montero RS, Espí PLL. Physical analysis of several organic signals for human echolocation: Oral vacuum pulses. Acta Acust united with Acust. 2009;95: 325–330. 10.3813/AAA.918155

[15] Thaler L, Reich GM, Zhang X, Wang D, Smith GE, Tao Z, et al. Mouth-clicks used by blind expert human echolocators–signal description and model based signal synthesis. PLOS Comput Biol. 2017;13. 10.1371/journal.pcbi.1005670 28859082 PMC5578488

[16] Thaler L, De Vos R, Kish D, Antoniou M, Baker C, Hornikx M. Human echolocators adjust loudness and number of clicks for detection of reflectors at various azimuth angles. Proc R Soc B Biol Sci. 2018;285. 10.1098/rspb.2017.2735 29491173 PMC5832709

[17] Pelegrín-García D, De Sena E, van Waterschoot T, Rychtáriková M, Glorieux C. Localization of a Virtual Wall by Means of Active Echolocation by Untrained Sighted Persons. 2018;139: 82–92. 10.1016/j.apacoust.2018.04.018

[18] Schenkman BN, Nilsson ME. Human echolocation: Blind and sighted persons’ ability to detect sounds recorded in the presence of a reflecting object. Perception. 2010;39: 483–501. 10.1068/p6473 20514997

[19] Pelegrín-García D, Rychtáriková M, Glorieux C. Single simulated reflection audibility thresholds for oral sounds in untrained sighted people. Acta Acust United with Acust. 2017;103: 492–505. 10.3813/AAA.919078

[20] Buchholz JM. A quantitative analysis of spectral mechanisms involved in auditory detection of coloration by a single wall reflection. Hear Res. 2011;277: 192–203. 10.1016/j.heares.2011.01.002 21236325

[21] Kolarik AJ, Scarfe AC, Moore BCJ, Pardhan S. Blindness enhances auditory obstacle circumvention: Assessing echolocation, sensory substitution, and visual-based navigation. PLoS One. 2017;12: 1–25. 10.1371/journal.pone.0175750 28407000 PMC5391114

[22] Thaler L, Castillo-Serrano J. People’s ability to detect objects using click-based echolocation: A direct comparison between mouth-clicks and clicks made by a loudspeaker. PLoS One. 2016;11: 1–14. 10.1371/journal.pone.0154868 27135407 PMC4852930

[23] Thaler L, Goodale MA. Echolocation in humans: an overview. Wiley Interdiscip Rev Cogn Sci. 2016;7: 382–393. 10.1002/wcs.1408 27538733

[24] Teng S, Whitney D. The acuity of echolocation: Spatial resolution in the sighted compared to expert performance. J Vis Impair Blind. 2011;105: 20–32. 21611133 PMC3099177

[25] Tim J. Beginner’s Guide to Ecolocation for the Blind and Visually Impaired. 2012.

[26] Beranek L, Martin DW. Concert & Opera Halls: How They Sound. J Acoust Soc Am. 1996;99: 2637–2637. 10.1121/1.414882

[27] Shtrepi L, Astolfi A, Pelzer S, Vitale R, Rychtáriková M. Objective and perceptual assessment of the scattered sound field in a simulated concert hall. J Acoust Soc Am. 2015;138: 1485–1497. 10.1121/1.4929743 26428786

[28] Shtrepi L, Astolfi A, D’Antonio G, Guski M. Objective and perceptual evaluation of distance-dependent scattered sound effects in a small variable-acoustics hall. J Acoust Soc Am. 2016;140: 3651–3662. 10.1121/1.4966267 27908082

[29] Shtrepi L, Pelzer S, Rychtáriková M, Vitale R, Astolfi A, Vorländer M. Objective and subjective assessment of scattered sound in a virtual acoustical environment simulated with three different algorithms. 41st International Congress and Exposition on Noise Control Engineering 2012. New York City, New York, USA: Institute of Noise Control Engineering; 2012. pp. 7394–7405.

[30] Vitale R. Perceptual aspects of sound scattering in concert halls. Logos Verlag Berlin GmbH; 2015.

[31] Torres RR, Kleiner M, Dalenbäck B-I. Audibility of diffusion on Room acoustics Auralization: an initial investigation. ACTA Acust United with Acust. 2000;86: 919–927.

[32] Brinkmann F, Aspöck L, Ackermann D, Lepa S, Vorländer M, Weinzierl S. A round robin on room acoustical simulation and auralization. J Acoust Soc Am. 2019;145: 2746–2760. 10.1121/1.5096178 31046379

[33] Calleri C, Astolfi A, Armando A, Shtrepi L. On the ability to correlate perceived sound to urban space geometries. Sustain Cities Soc. 2016;27: 346–355. 10.1016/j.scs.2016.05.016

[34] Robinson PW, Pätynen J, Lokki T, Suk Jang H, Yong Jeon J, Xiang N. The role of diffusive architectural surfaces on auditory spatial discrimination in performance venues. J Acoust Soc Am. 2013;133: 3940–3950. 10.1121/1.4803846 23742348

[35] Robinson PW, Walther A, Faller C, Braasch J. Echo thresholds for reflections from acoustically diffusive architectural surfaces. J Acoust Soc Am. 2013;134: 2755–2764. 10.1121/1.4820890 24116414

[36] Claes N, Zelem L, Pelegrín-García D, Rychtarikova M, Glorieux C. Echolocation Recognition of Different Wall Textures. Proceedings of EuroRegio 2016. Porto, Portugal; 2016.

[37] de Vos R, Hornikx M. Acoustic Properties of Tongue Clicks used for Human Echolocation. Acta Acust united with Acust. 2017;103: 1106–1115. 10.3813/AAA.919138

[38] Pelegrín-García D, Rychtáriková M, Glorieux C. Audibility thresholds of a sound reflection in a classical human echolocation experiment. Acta Acust united with Acust. 2016;102: 530–539. 10.3813/AAA.918970

[39] De Vos R, Hornikx M. Human ability to judge relative size and lateral position of a sound reflecting board using click signals: Influence of source position and click properties. Acta Acust united with Acust. 2018;104. 10.3813/AAA.919153

[40] Zahorik P, Brungart DS, Bronkhorst AW. Auditory distance perception in humans: A summary of past and present research. Acta Acust united with Acust. 2005;91: 409–420.

[41] Hugo Fast HZ. PsychoAcoustics—Facts and Models. Springer. 2007.

[42] Boullet I. La sonie des sons impulsionnels: perception, mesures et modèles. 2005.

[43] Genesis S.A. Loudness Online. 2018 [cited 20 Apr 2018]. Available: http://www.genesis-acoustics.com/cn/%5Cnindex.php?page=32.

[44] Advanced CAE Research. FastBEM Acoustics 3.0 User Guide. 2012.

[45] Paulsen RR, Bærentzen JA, Larsen R. Markov random field surface reconstruction. IEEE Trans Vis Comput Graph. 2010;16: 636–646. 10.1109/TVCG.2009.208 20467061

[46] Bernschütz B. A Spherical Far Field HRIR/HRTF Compilation of the Neumann KU 100. Proceedings of the 40th Italian (AIA) annual conference on acoustics and the 39th German annual conference on acoustics (DAGA) conference on acoustics. 2013. pp. 592–595.

[47] Cabrera D, Sato H, Martens WL, Lee D. Binaural measurement and simulation of the room acoustical response from a person’s mouth to their ears. Acoust Aust. 2009;37: 98–103.

[48] Poulsen T. Psychoacoustic Measuring Methods. Lect note no 3108-e, Lyngby, Denmark Osted-DTU, Acoust Technol. 2002.

[49] Kennedy JJ. Gade, 1968; Bruning, 1968; Kennedy, 1969),. 1970; 885–889. 10.1186/1471-2334-10-37 20178619 PMC2843612

[50] Cohen J. Eta-squared and partial eta-squared in fixed factor ANOVA designs. Educ Psychol Meas. 1973;33: 107–112.

[51] Norouzian R, Plonsky L. Eta- and partial eta-squared in L2 research: A cautionary review and guide to more appropriate usage. Second Lang Res. 2018;34: 257–271. 10.1177/0267658316684904

[52] Levine TR, Hullett CR. Eta Squared, Partial Eta Squared, and Misreporting of Effect Size in Communication Research. Hum Commun Res. 2002;28: 612–625. 10.1093/hcr/28.4.612

[53] Wallmeier L, Geßele N, Wiegrebe L. Echolocation versus echo suppression in humans. Proc Biol Sci. 2013;280. 10.1098/rspb.2013.1428 23986105 PMC3768302

[54] Shtrepi L, Astolfi A, Puglisi GE, Masoero MC. Effects of the distance from a diffusive surface on the objective and perceptual evaluation of the sound field in a small simulated variable-acoustics hall. Appl Sci. 2017;7. 10.3390/app7030224

[55] Shtrepi L. Investigation on the diffusive surface modeling detail in geometrical acoustics based simulations. J Acoust Soc Am. 2019;145: EL215–EL221. 10.1121/1.5092821 31067969

[56] Rice CE, Feinstein SH, Schusterman RJ. Echo-detection ability of the blind: Size and distance factors. J Exp Psychol. 1965;70: 246–251. 10.1037/h0022215 14343251

[57] Wallmeier L, Wiegrebe L. Ranging in human sonar: Effects of additional early reflections and exploratory head movements. PLoS One. 2014;9: 1–28. 10.1371/journal.pone.0115363 25551226 PMC4281102

[58] Schörnich S, Nagy A, Wiegrebe L. Discovering your inner bat: Echo-acoustic target ranging in humans. JARO—J Assoc Res Otolaryngol. 2012;13: 673–682. 10.1007/s10162-012-0338-z 22729842 PMC3441954

[59] Tonelli A, Brayda L, Gori M. Depth echolocation learnt by novice sighted people. PLoS One. 2016;11: 1–14. 10.1371/journal.pone.0156654 27257689 PMC4892586

[60] Rychtáriková M, Bogaert T Van Den, Vermeir G, Wouters J. Perceptual validation of virtual room acoustics: Sound localisation and speech understanding. Appl Acoust. 2011;72: 196–204. 10.1016/j.apacoust.2010.11.012

